# Effectiveness of a participatory educational intervention on menstrual health and hygiene knowledge, attitudes, and practices among nursing and midwifery students in Bangladesh

**DOI:** 10.1371/journal.pone.0362603

**Published:** 2026-11-10

**Authors:** Md Nahid Uz Zaman, Akira Sai, Taro Yamauchi

**Affiliations:** 1 Graduate School of Health Sciences, Hokkaido University, Sapporo, Hokkaido, Japan; 2 Directorate General of Nursing and Midwifery, Ministry of Health and Family Welfare, Dhaka, Bangladesh; 3 Global Station for Indigenous Studies and Cultural Diversity, Hokkaido University, Sapporo, Hokkaido, Japan; 4 Faculty of Health Sciences, Hokkaido University, Sapporo, Hokkaido, Japan; Cranfield University, UNITED KINGDOM OF GREAT BRITAIN AND NORTHERN IRELAND

## Abstract

**Background:**

Menstrual Health and Hygiene (MHH) is fundamental to women’s well-being but remains underprioritized in professional health education. In Bangladesh, nursing and midwifery students, future frontline health workers, rarely receive structured menstrual health curricula. This study assessed the effectiveness of a Participatory Educational (PE) intervention in improving menstrual Knowledge, Attitudes, and Practices (KAP) among nursing students.

**Methods:**

A quasi-experimental pre-test–post-test study was conducted among 384 female nursing and midwifery students in Rajshahi, Bangladesh, with 192 students each in the intervention and control groups. The four-week PE intervention, adapted from WaterAid’s *Menstrual Hygiene Matters* guide, covered menstrual physiology, hygiene practices, stigma reduction, and Water, Sanitation and Hygiene (WASH) management. The post-intervention evaluation was conducted six months after the intervention. Data were collected using a pre-tested structured questionnaire. Knowledge and practice indicators were analyzed using McNemar’s and chi-square tests, while attitude subscale scores were analyzed using paired and independent samples t-tests, with p < 0.05 considered statistically significant.

**Results:**

Baseline socio-demographic characteristics were broadly comparable between groups. At endline, the intervention group showed greater improvement than the control group in several knowledge indicators, including correct identification of average menstrual cycle length (78% vs. 34%, p < 0.001) and reproductive organs (65% vs. 28%, p < 0.001). Attitudes toward menstruation also improved, particularly in perceiving menstruation as a natural event (mean 5.9 vs. 5.1, p < 0.001) and for preparedness and predictability of menstruation (mean 6.0 vs. 4.8, p < 0.001); both were measured on a 1–7 scale. Several practices improved in the intervention group, including consistent use of disposable sanitary pads (88% vs. 58%, p < 0.001), changing menstrual materials at least three times daily (65% vs. 39%, p < 0.001), and handwashing before changing materials (76% vs. 55%, p < 0.001).

**Conclusion:**

A short PE intervention, improved selected MHH-related KAP among nursing and midwifery students. Given the single-site quasi-experimental design and the use of only one post-intervention evaluation at six months, the sustainability and trajectory of these changes over time remain uncertain. Multi-site studies with repeated follow-up assessments are needed to confirm these findings and assess their broader applicability to tertiary nursing and midwifery education.

**Clinical trial number:** not applicable.

## 1. Introduction

Menstruation is a universal biological process and a vital indicator of reproductive and overall health for nearly half of the world’s population. Despite its normalcy, menstruation remains shrouded in social silence, stigma, and misinformation across many societies, especially in Low- and Middle-Income Countries (LMICs) [1,2]. Poor Menstrual Health and Hygiene (MHH) management has been linked not only to reproductive tract infections but also to school absenteeism, psychosocial distress, and gender-based inequalities [3–5]. Addressing MHH is therefore a public health priority directly connected to the Sustainable Development Goals (SDGs), particularly SDG 3 (Good Health and Well-being), SDG 4 (Quality Education), SDG 5 (Gender Equality), and SDG 6 (Clean Water and Sanitation) [6]. Although global awareness of menstrual health has grown substantially over the last decade, research and interventions have been largely concentrated on adolescent schoolgirls, while older adolescents and adult women, especially those in vocational and healthcare training remain neglected in both research and policy frameworks [7,8]. The World Health Organization [3] and WaterAid [9]have emphasized that ensuring menstrual dignity requires not only access to sanitary materials and facilities but also education that builds Knowledge, Attitudes, and Practice (KAP) to manage menstruation hygienically and without shame.

In South and Southeast Asia, Menstrual Hygiene Management (MHM) continues to be influenced by traditional taboos, gender norms, and infrastructural inadequacies. Studies in Nepal, Bangladesh, and India indicate that menstruation is often surrounded by secrecy and culturally embedded notions of impurity, which may contribute to restrictions on women’s diet, mobility, and participation in daily activities [10–12]. Many young women lack comprehensive knowledge of menstrual physiology prior to menarche and rely on peers or family members who often hold misconceptions themselves. Inadequate Water, Sanitation, and Hygiene (WASH) facilities in educational institutions exacerbate these challenges by making MHM unhygienic and stressful [13]. Bangladesh, in particular, faces a dual challenge. While school-based MHH educational programs have been implemented through partnerships with UNICEF and WaterAid, few initiatives have extended into tertiary or professional education. Research shows that even university and nursing students who are expected to possess health literacy often lack accurate information and experience menstrual stigma [14–16]. Within nursing education settings in Bangladesh, cultural norms surrounding menstruation may discourage open discussion in both academic environments and student hostels, which provide residential accommodation for students, potentially reinforcing secrecy and shame [15]. Such barriers may limit students’ opportunities to develop knowledge and confidence in managing their own menstrual health and in addressing menstrual health concerns in their future professional practice.

Despite recent progress in menstrual health research, a significant gap persists in understanding how structured educational interventions affect menstrual KAP among nursing and midwifery students. Existing literature largely focuses on adolescents, overlooking the specific needs of young adult women who are preparing for healthcare professions [17,18]. As frontline healthcare providers, nurses and midwives play a key role in reproductive health education and community health promotion in Bangladesh. Therefore, improving menstrual health knowledge, attitudes, and practices (KAP) among nursing and midwifery students is important not only for their personal well-being but also for strengthening their capacity to provide accurate and empathetic care to patients. While they follow distinct clinical tracks, their foundational coursework, hostel environments, and exposure to community health topics are similar, and both disciplines will care for menstruating clients in clinical and community settings. Nursing and midwifery students are an important population for menstrual health research; however, evidence on educational interventions addressing menstrual health and hygiene among these students in Bangladesh remains limited [19]. As future providers and educators, they will be expected to promote hygiene and reproductive well-being. However, internalized stigma and misinformation can undermine both personal well-being and professional practice [20]. Studies from various settings indicate that myths about menstruation persist even among health-professional students, and discussions often remain limited due to cultural discomfort [19,21,22] Improving MHH literacy among nursing and midwifery students has potential “multiplier” effects [15,19]: better-informed providers are more likely to give accurate, empathetic counseling to patients and to support community-level health promotion. While evidence directly suggested long-term community impacts is still limited, interventions in related areas of sexual and reproductive health show that provider education can improve quality of counseling and client outcomes. Embedding menstrual health education into curricula is therefore consistent with contemporary emphasis on gender-sensitive, holistic care [23,24].

Despite increasing attention to MHH in educational settings, evidence on structured PE interventions targeting nursing and midwifery students remains limited. This study aimed to evaluate the effectiveness of a structured PE intervention on MHH related KAP among female nursing and midwifery students in Bangladesh. Focusing on students residing in a residential nursing college provided an opportunity to evaluate how participatory education can influence MHH behaviors within both academic and shared living environments.

## 2. Methodology

### 2.1 Study design

This study employed a quasi-experimental pre-test and post-test design with intervention and control groups to evaluate changes MHH related KAP associated with a structured Participatory Educational (PE) intervention among nursing and midwifery students in Bangladesh. Random assignment of individual students to the intervention and control groups was not undertaken because the intervention was delivered within existing class structures and individual-level allocation could have increased the potential for information exchange between groups. Instead, existing class sections were allocated to either the intervention or control condition. Therefore, the study remained quasi-experimental rather than a randomized controlled trial. Stratified random sampling was used separately for participant selection and did not determine intervention allocation. Students were stratified by academic year, and participants were randomly selected within each stratum from official class attendance registers. This approach was used to obtain representation across academic years rather than to randomize exposure to the intervention [18,25,26]. The intervention was guided by the principles of participatory learning, gender-sensitive health promotion, and adult education theory, ensuring active student engagement and long-term retention of menstrual health knowledge. The intervention model was structured around three interrelated learning domains cognitive, affective, and behavioral pathways adapted from Bloom’s taxonomy and social cognitive learning theory [27–29].

### 2.2 Theoretical framework

Effective PE interventions rely on understanding how individuals acquire and apply knowledge. This study draws on Bloom’s taxonomy and Bandura’s social cognitive theory, which together emphasize cognitive, affective, and behavioral pathways of learning [27–29]. The cognitive pathway focuses on factual knowledge and understanding of menstrual physiology and hygiene, addressed through interactive lectures and demonstrations [27,30]. The affective pathway targets emotions, beliefs, and values, aiming to reduce shame and normalize menstruation via group discussion, reflection, and supportive dialogue [29,31]. The behavioral pathway strengthens self-efficacy and practical skills through role-play, problem-solving, and practice in applying hygienic behaviors [28,32]. This framework is operationalized through the *Menstrual Hygiene Matters* training model [9,33], which emphasizes participatory learning, peer engagement, and sensitivity to local WASH constraints. Such an approach has proven effective in diverse contexts from schoolgirls in Kenya [4], but has rarely been tested within professional healthcare education [8]. The present intervention adapted this model to the realities of Bangladeshi hostel-based nursing and midwifery students, focusing on what students could realistically implement in their daily lives and future practice. The conceptual framework is shown in Fig 1. The intervention design was also informed by the socio-cultural environment of nursing college hostels, where menstruation is often treated as a private or sensitive topic and open discussion among students may be limited. Such cultural silence can reinforce misinformation and discourage help-seeking behaviors. To address this context, the educational program incorporated participatory and peer-based learning strategies that created safe spaces for discussion, reflection, and experience-sharing. Small-group dialogue, role-play activities, and peer engagement were intentionally included to reduce embarrassment and normalize conversations about menstruation within the hostel community. By aligning the educational approach with the social realities of students’ living environments, the intervention aimed not only to improve knowledge but also to transform attitudes and communication patterns surrounding menstrual health.

**Fig 1:**
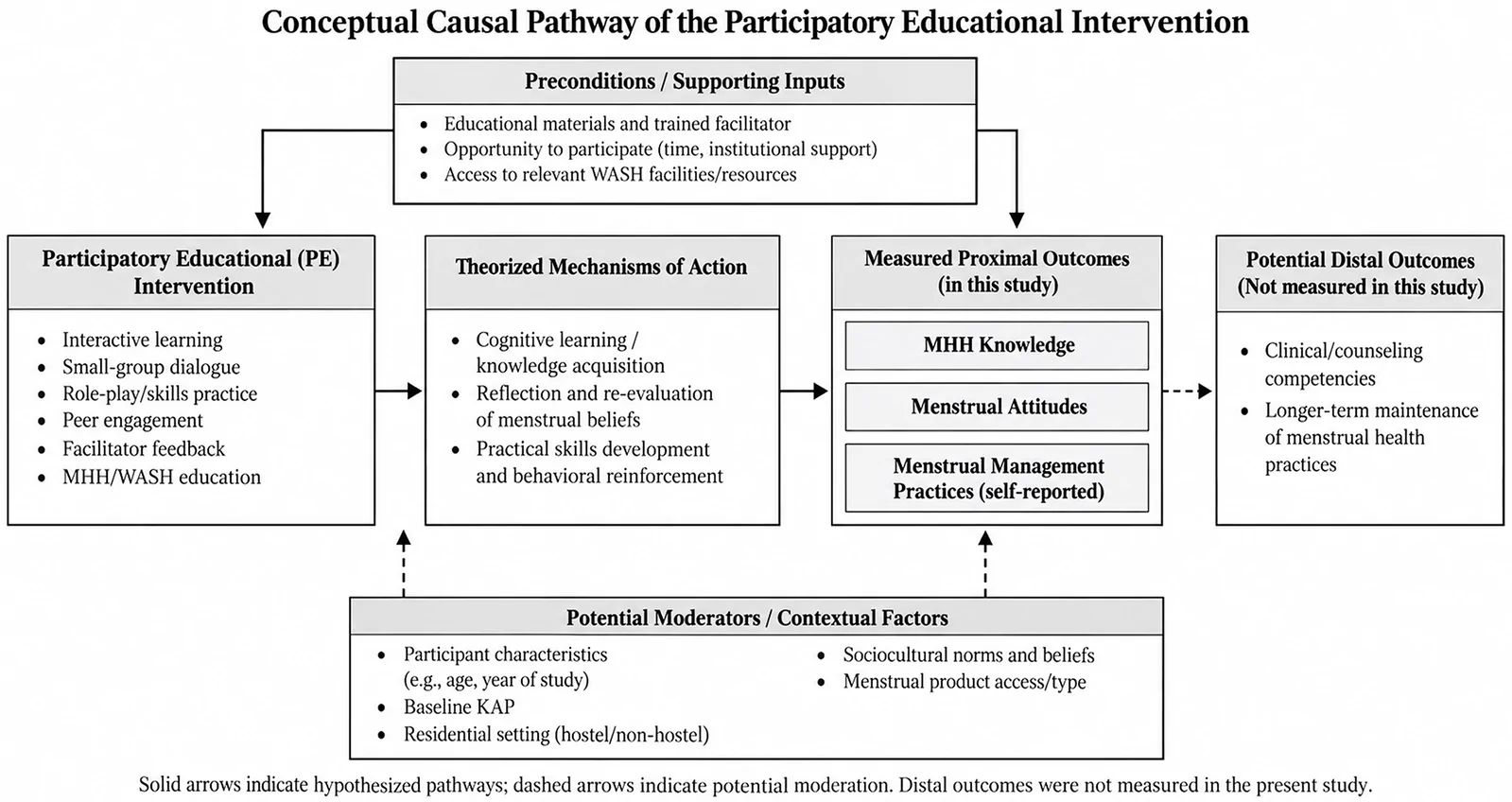
Theoretical framework.

### 2.3 Study setting, population and sampling procedures

The study was conducted between November 2024 and August 2025 at RNC, one of the largest government nursing institutions in northwestern Bangladesh, which enrolls students from diverse socioeconomic and geographic backgrounds, including both urban and rural communities. RNC was selected because it is a government institution operating under the nationally standardized curriculum of the Bangladesh Nursing and Midwifery Council (BNMC). As a government nursing institution following the standardized curriculum and admission procedures of the BNMC, the college enrolls students from multiple districts and socio-economic backgrounds, making it reasonably comparable to other public nursing colleges in Bangladesh. Institutional approval and logistical feasibility for implementing a structured educational intervention also influenced site selection. The selection of RNC was additionally informed by findings from our previous multicenter study conducted across seven nursing colleges located in four districts and three geographic regions of Bangladesh. That earlier study reported that nursing students shared broadly comparable MHH challenges across institutional settings, including gaps in menstrual knowledge, stigma-related experiences, and WASH-related barriers [15,19]. These findings supported the suitability of RNC as a feasible and contextually relevant site for evaluating an educational intervention.

The study population included all female undergraduate nursing and midwifery students enrolled at the college who had experienced menarche and provided written informed consent. To ensure representation across academic levels, stratified random sampling was used for participant selection. Students were stratified by academic year (first through fourth year), and eligible participants were randomly selected within each stratum from official class attendance registers. This random sampling procedure determined which students were included in the study and did not determine allocation to the intervention or control condition. Each academic-year class consisted of two existing sections; one section was allocated to the intervention condition and the other to the control condition. Section-level allocation was used to preserve existing class structures and reduce, although not eliminate, the potential for information exchange between intervention and control participants. To reduce potential systematic differences between classes, baseline comparisons were conducted to examine equivalence in socio-demographic characteristics and baseline MHH indicators between the intervention and control groups. Students who were seriously ill or absent during both baseline and endline data collection were excluded because incomplete participation could compromise the assessment of intervention effects. Pregnant students were also excluded due to the possibility that pregnancy-related physiological and psychosocial changes could influence menstrual experiences, attitudes, and hygiene practices independently of the intervention, potentially affecting comparability of outcomes. Students were approached through announcements made during scheduled class sessions. Participation was entirely voluntary and no financial or academic incentives were provided. Students who expressed interest were invited to participate and were enrolled after providing written informed consent. Prior to enrollment, students were briefed in small groups about the study objectives, procedures, potential risks, and their right to withdraw at any time without academic consequences. Confidentiality was ensured by assigning unique identification codes and by administering questionnaires anonymously. Data were stored securely and no personal identifiers were included in the dataset, which was particularly important in the shared hostel environment (Fig 2).

**Fig 2:**
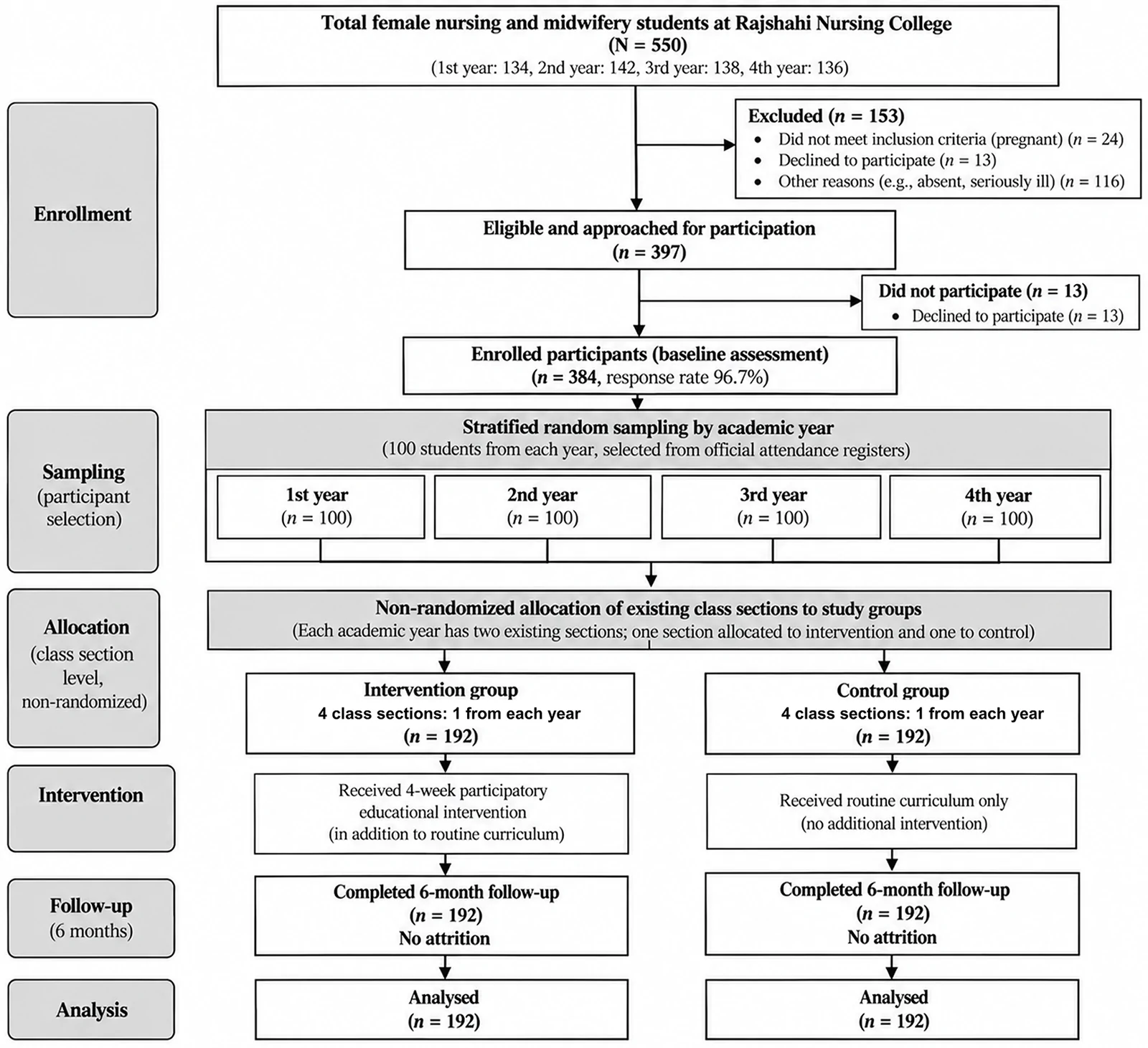
Flow diagram of participant recruitment, allocation, follow-up, and analysis.

### 2.4 Sample size determination

The sample size was determined using G*Power version 3.1 (Heinrich Heine University, Dusseldorf, Germany) software for a two-tailed independent samples t-test. Based on prior studies of menstrual and reproductive health interventions, an expected small-to-medium effect size (Cohen’s d = 0.3), alpha level of 0.05, and statistical power of 80% were applied [18,26]. The minimum required sample size was calculated as 176 participants per group. To account for potential non-response or attrition, a 10% adjustment was made, resulting in 192 participants in the intervention group and 192 in the control group, for a total of 384 students.

Allocating participants by class section preserved the natural learning structure and minimized logistical disruption, while pre-test comparisons confirmed baseline equivalence between groups in socio-demographic characteristics and MHH indicators. The final sample size provided sufficient statistical power to detect meaningful improvements in KAP outcomes following the intervention, consistent with standards for educational intervention trials in LMIC contexts [18,25,34]

### 2.5 Data collection instruments

Data were collected using a pre-tested structured questionnaire that was developed by adapting items from several internationally recognized instruments. The knowledge domain was adapted from previous study [12], which assesses menstrual physiology, hygiene practices, and product use in school-based interventions. The attitudinal domain was derived from the Menstrual Attitude Questionnaire (MAQ) originally developed by Brooks-Gunn and Ruble [35], which captures multidimensional beliefs about menstruation as a natural, debilitating, or bothersome event. The MAQ items were culturally adapted to the Bangladeshi context through expert review and pilot testing, with minor modifications in wording to ensure relevance to local social norms surrounding menstruation while maintaining the conceptual integrity of the original scale. Practice-related items, including access, comfort, and adequacy of menstrual management behaviors, were adapted from the Menstrual Practice Questionnaire (MPQ) [36].

The final questionnaire comprised four sections: (i) socio-demographic characteristics; (ii) menstrual knowledge (nine items); (iii) attitude (33 items across five MAQ sub-domains); and (iv) hygienic practices (28 items) (*see Supplementary* S1 File). Responses were collected using a combination of multiple-choice and five-point Likert-scale items. To ensure content validity and contextual appropriateness, the instrument was reviewed by three experts in public health nursing and women’s health. A pilot test was conducted with 30 nursing students from another college to assess clarity, comprehension, and reliability. The instrument showed satisfactory internal consistency, with Cronbach’s α = 0.84 for the total scale and domain-specific reliabilities of 0.80 for knowledge, 0.82 for attitude, and 0.79 for practice, indicating good reliability and stability across constructs. During pilot testing, students were invited to provide feedback on the clarity, cultural relevance, and sensitivity of questionnaire items. Minor wording adjustments were made to ensure that questions reflected locally understood terminology and menstrual practices in the Bangladeshi context. Construct validity was supported by aligning questionnaire domains with established theoretical constructs of menstrual KAP. Items were selected from previously validated instruments and reviewed by subject experts to ensure conceptual consistency with the study objectives.

### 2.6 Description of the intervention

The educational intervention was designed and implemented following the WaterAid “Menstrual Hygiene Matters: Training Guide for Practitioners” [9,33], a globally recognized resource that situates menstrual health within the broader WASH and gender-equity framework. The program emphasized PE, self-efficacy enhancement, and stigma reduction.

The intervention comprised four weekly face-to-face sessions of 60–90 minutes each, delivered over one month. A combination of lectures, interactive discussions, role-plays, visual demonstrations, and short documentary films including Adolescent School Girls in Bangladesh on Managing Menstruation and Making the Invisible Visible (WaterAid India/Vatsalya) was used to maximize engagement and retention. Session 1 introduced reproductive anatomy and physiology, explained the menstrual cycle, and dispelled common myths and misconceptions. Session 2 covered menstrual hygiene materials both disposable and reusable highlighting proper usage, washing, drying, and safe disposal practices. Session 3 addressed stigma reduction, effective communication, and peer-support strategies to normalize menstruation in academic and clinical environments. Session 4 focused on WASH facility management, access to clean water and soap, and the creation of a supportive institutional environment for menstruating students (Table 1).

**Table 1:** Content and delivery of the participatory educational intervention.

Session	Objective	Main topics	Participatory activities	Duration
1. Understanding menstruation	Strengthen understanding of menstrual physiology	Reproductive anatomy; menstrual cycle; normal menstruation; myths and misconceptions	Interactive lecture, visual demonstration, group discussion, question-and-answer session	60–90 min
2. Menstrual management practices	Develop practical menstrual-management knowledge and skills	Disposable and reusable products; changing practices; washing and drying reusable materials; hand hygiene; disposal; pain-management strategies	Product demonstration, problem-solving activities, small-group discussion, practical demonstration	60–90 min
3. Stigma, communication, and peer support	Encourage reflection on menstrual beliefs and communication	Menstrual stigma; myths; communication; peer support; discussing menstruation in academic and clinical settings	Small-group dialogue, role-play, reflection, peer sharing	60–90 min
4. WASH and supportive environments	Strengthen understanding of environmental requirements for menstrual management	Water and soap; privacy; toilets; disposal facilities; menstrual management in student residential and educational settings	Case discussion, problem-solving, visual/documentary materials, group discussion	60–90 min

Each participant in the intervention group received printed handouts, a menstrual-cycle tracking calendar, and sample menstrual products for demonstration purposes. The educational materials included illustrated handouts on menstrual physiology, the normal menstrual cycle, common myths and misconceptions, hygienic use of disposable and reusable menstrual products, handwashing, cleaning and drying of reusable materials, safe disposal of used menstrual materials, pain-management strategies, menstrual tracking, and ways to communicate about menstruation without shame. Sessions were facilitated by the first author, a registered nurse from Bangladesh, along with female nurse educators and a gynecologist. To ensure consistency across sessions, a standardized training outline and facilitator guide were used. All facilitators received prior orientation on the intervention content, session objectives, and participatory teaching methods to maintain uniform delivery across sessions. Participants in the control group were not informed about the timing or specific content of the training during the study period in order to minimize expectation bias that could influence responses. To maintain ethical fairness, all educational materials were subsequently provided to the control group after the completion of the final data collection.

### 2.7 Data collection procedure

Baseline data were collected from both groups one week before the intervention, and follow-up (endline) data were collected six months after completion of the four-week intervention. The six-month follow-up interval was selected to assess whether differences in MHH-related KAP were observable beyond the immediate post-intervention period rather than capturing only short-term responses to the educational sessions. The same questionnaire was administered at both time points to ensure comparability. Data collection was supervised by the first author and trained research assistants who were not involved in delivering the intervention. Questionnaires were self-administered, and supervisors provided standardized procedural instructions without influencing participants’ responses. All participants were briefed on the objectives and assured of anonymity and confidentiality. Attendance was recorded at each of the four intervention sessions using participant identification codes. Students who missed any session were contacted through class representatives and were offered a brief make-up discussion using the same educational materials before the next session. Follow-up data collection was conducted during scheduled class hours, which helped ensure full participation at endline. No attrition occurred between baseline and endline assessments; all 384 enrolled participants completed both surveys. Attendance records confirmed that all intervention-group participants attended the full intervention cycle either through regular sessions or make-up discussions. Unique identification codes were assigned to match each participant’s pre- and post-test responses. Questionnaires were self-administered in classroom settings under direct supervision to ensure uniformity, minimize discussion among participants, and prevent missing responses. At the end of each data-collection day, completed questionnaires were reviewed for completeness before being securely stored in locked cabinets for data entry.

### 2.8 Data management and statistical analysis

All data were entered and analyzed using JMP Pro 18 (SAS Institute Inc., USA). The allocation to the intervention and control conditions occurred at the existing class-section level, the primary analyses were conducted at the individual-student level and did not explicitly adjust standard errors for clustering by class section. Descriptive statistics were used to summarize participants’ socio-demographic characteristics and study variables. Continuous variables were presented as means and standard deviations (SD), whereas categorical variables were presented as frequencies and percentages. Baseline comparability between the intervention and control groups was assessed by examining the magnitude and precision of differences. For continuous variables, mean differences with 95% confidence intervals (CI) and standardized mean differences (SMD) were calculated. For categorical variables, proportion differences with 95% CI were reported. Knowledge and practice items were analyzed as categorical variables. For items requiring a specific correct response, such as average menstrual cycle length, responses were recoded into binary categories (correct vs. incorrect/don’t know) for analysis. Within-group changes from baseline to endline for individual knowledge and practice indicators were assessed using McNemar’s test. Between-group comparisons at endline were conducted using chi-square tests, and proportion differences with 95% CI were reported. The 33 MAQ items were grouped into five subscales: menstruation as a debilitating event (7 items), menstruation as a bothersome event (6 items), menstruation as a natural event (5 items), anticipation and prediction of menstruation (7 items), and denial of any effect of menstruation (8 items). For each subscale, item responses were averaged to generate a mean domain score. Negatively worded items were reverse coded before analysis so that higher values consistently represented more positive attitudes toward menstruation. Within-group changes in MAQ subscale scores were examined using paired t-tests, while between-group differences at the endline were assessed using independent samples t-tests. Mean differences with 95% CI were calculated, and Cohen’s d was used to estimate the magnitude of pre–post changes in the intervention group. All statistical tests were two-sided, and statistical significance was set at p < 0.05. The KAP outcomes were predefined based on the study questionnaire and intervention content. No formal adjustment for multiple comparisons was applied; therefore, the possibility of type I error cannot be excluded. P-values were interpreted alongside effect estimates and 95% confidence intervals.

### 2.9 Ethical approval and consent to participate

Ethical approval was obtained from the Ethical Review Committee of the Faculty of Health Sciences, Hokkaido University, Japan (23–52). Institutional permission was also secured from Directorate general of Nursing and Midwifery, Ministry of Health and Family Welfare Bangladesh (45.03.0000.008.14.097.2021-650). All participants were informed about the study objectives, voluntary participation, and the right to withdraw without penalty. Written informed consent was obtained prior to participation. Data confidentiality was maintained through anonymized coding and restricted data access. The educational materials used in this intervention were adapted from publicly available, open-access resources (e.g., WaterAid), in accordance with their Creative Commons licensing terms with full attribution to the original sources. The study strictly adhered to the ethical standards outlined in the Declaration of Helsinki for research involving human participants.

## 3. Results

A total of 397 eligible students were approached, of whom 384 participated and completed the baseline assessment, corresponding to a response rate of 96.7%. Thirteen students declined participation, primarily for personal reasons or lack of interest.

### 3.1 Socio-demographic characteristics of participants

A total of 384 female nursing and midwifery students participated in the study, with 192 participants in each of the intervention and control groups. Baseline characteristics were examined for potential between-group imbalance. A small difference in mean age was observed between the intervention and control groups (22.5 ± 3.2 vs. 23.1 ± 2.7 years, respectively; mean difference = −0.6 years, 95% CI: −1.19 to −0.01; SMD = −0.20; p = 0.048). Given the small magnitude of this difference, age was not included as a covariate in the primary unadjusted analyses. Nevertheless, residual confounding by age cannot be excluded and was considered when interpreting the findings. Most participants were nursing students (84% and 89%, respectively), and approximately three-quarters were from rural backgrounds. Overall, baseline socio-demographic characteristics were broadly comparable between the intervention and control groups. Detailed participant characteristics are presented in Table 2 (Data are presented as mean ± SD or n (%), as appropriate. Differences are calculated as intervention minus control. SMD = standardized mean difference; CI = confidence interval; BDT = Bangladeshi taka. †Other religion includes participants reporting a religion other than Islam or Hinduism.).

**Table 2:** Socio-demographic characteristics of study participants (N = 384).

Variable	Control(n = 192)	Intervention(n = 192)	Difference(Intervention − Control)	95% CI	SMD	p-value
Age (years), mean ± SD	23.1 ± 2.7	22.5 ± 3.2	−0.6	−1.19 to −0.01	−0.20	0.048
Age at menarche (years), mean ± SD	12.9 ± 1.5	13.0 ± 1.3	0.1	−0.18 to 0.38	0.07	0.486
**Student category, n (%)**						
Nursing	170 (89%)	161 (84%)	−4.7%	−11.6% to 2.2%	−0.14	0.183
Midwifery	22 (11%)	31 (16%)	4.7%	−2.2% to 11.6%	0.14	0.183
**Religion, n (%)**						
Islam	182 (95%)	169 (88%)	−6.7%	−13.2% to −0.3%	−0.24	0.060
Hinduism	8 (4%)	19 (10%)	5.7%	0.7% to 10.8%	0.23	
Other†	2 (1%)	4 (2%)	1.0%	−1.4% to 3.5%	0.08	
**Father’s education, n (%)**						
≥ Secondary	57 (30%)	62 (32%)	2.6%	−6.6% to 11.9%	0.06	0.581
<Secondary	135 (70%)	130 (68%)	−2.6%	−11.9% to 6.6%	−0.06	0.581
**Mother’s education, n (%)**						
≥ Secondary	73 (38%)	78 (41%)	2.6%	−7.2% to 12.4%	0.05	0.601
<Secondary	119 (62%)	114 (59%)	−2.6%	−12.4% to 7.2%	−0.05	0.601
**Residence, n (%)**						
Hostel	145 (76%)	146 (76%)	0.5%	−8.0% to 9.1%	0.01	0.905
Outside hostel	47 (24%)	46 (24%)	−0.5%	−9.1% to 8.0%	−0.01	0.905
**Birthplace, n (%)**						
Rural	149 (78%)	144 (75%)	−2.6%	−11.1% to 5.9%	−0.06	0.548
Urban	43 (22%)	48 (25%)	2.6%	−5.9% to 11.1%	0.06	0.548
**Monthly family income, n (%)**						
≤ 20,000 BDT	115 (60%)	122 (63%)	3.6%	−6.1% to 13.4%	0.08	0.462
> 20,000 BDT	77 (40%)	70 (37%)	−3.6%	−13.4% to 6.1%	−0.08	0.462

### 3.2 Changes in knowledge on menstrual health and hygiene

Table 3 summarizes pre- and post-intervention knowledge outcomes. At baseline, both groups showed moderate awareness of menstrual physiology and hygiene. Following the educational intervention, the intervention group exhibited substantial improvement in nearly all knowledge indicators, while the control group showed minimal or no change. Some changes were largely attributable to classroom exposure rather than targeted training, however. For example, correct identification of the average menstrual cycle length increased from 8% at baseline to 78% at endline in the intervention group, compared with 26% to 34% in the control group. At endline, this corresponded to a 44.3 percentage-point higher prevalence in the intervention group than in the control group (95% CI: 35.4 to 53.2; p < 0.001). Likewise, identification of the female reproductive organs improved from 35% to 65% in the intervention group, compared with 20% to 28% in the control group, with an endline difference of 37.0 percentage points (95% CI: 27.7 to 46.2; p < 0.001). Knowledge of uterine shedding as the cause of menstruation showed a smaller improvement, increasing from 22% to 34% in the intervention group, with an endline between-group difference of 9.9 percentage points (95% CI: 0.9 to 18.9; p = 0.032). Knowledge regarding washing reusable menstrual materials with soap improved from 33% to 53% in the intervention group, whereas little change was observed in the control group (54% to 52%), although the endline difference between groups was small and not statistically significant (difference: 1.0 percentage point; 95% CI: −8.9 to 11.0; p = 0.838). Proper disposal practices and knowledge of recommended changing frequency showed only limited improvement, suggesting that some concepts may require repeated reinforcement beyond a short educational intervention. In the Table 3, Values are presented as n (%) of participants providing the prespecified reference/correct response. Absolute change is expressed as the percentage-point (pp) difference between baseline and endline within each group. Within-group changes were assessed separately for the intervention and control groups using McNemar’s test for paired binary responses. Between-group comparisons at endline were assessed using chi-square tests, with absolute differences in proportions and 95% confidence intervals (CI) reported. For the age-at-menarche item, 13 years was used as the prespecified reference response for the average age at menarche; this should not be interpreted as indicating that menarche at other ages is abnormal or incorrect.

**Table 3:** Knowledge of menstrual health and hygiene among participants.

Knowledge indicator	Control baseline n (%)	Control endline n (%)	Control change (pp)	Illustrative assumed Control McNemar p	Intervention baseline n (%)	Intervention endline n (%)	Intervention change (pp)	Reported Intervention McNemar p	Endline difference	95% CI	Between-group p
Identification of 13 years as the reference average age at menarche	42 (22%)	117 (61%)	+39.1	<0.001	38 (20%)	106 (55%)	+35.4	<0.05	−5.7%	−15.6% to 4.1%	0.255
Correct understanding of menstrual bleeding as shedding of the uterine lining	46 (24%)	46 (24%)	0.0	1.000	42 (22%)	65 (34%)	+12.0	<0.05	9.9%	0.9% to 18.9%	0.032
Correct identification of average menstrual cycle length (28 days)	50 (26%)	65 (34%)	+7.8	<0.05	15 (8%)	150 (78%)	+70.3	<0.05	44.3%	35.4% to 53.2%	<0.001
Correct identification of typical menstrual bleeding duration (3–7 days)	163 (85%)	163 (85%)	0.0	1.000	165 (86%)	165 (86%)	0.0	1.000	1.0%	−6.0% to 8.1%	0.772
Correct identification of reproductive organs	38 (20%)	54 (28%)	+8.3	<0.05	67 (35%)	125 (65%)	+30.2	<0.001	37.0%	27.7% to 46.2%	<0.001
Knowledge of ≥3 pain-relief methods	63 (33%)	100 (52%)	+19.3	<0.001	60 (31%)	75 (39%)	+7.8	<0.05	−13.0%	−22.9% to −3.1%	0.010
Correct knowledge of washing reusable menstrual materials with soap	104 (54%)	100 (52%)	−2.1	>0.05	63 (33%)	102 (53%)	+20.3	<0.05	1.0%	−8.9% to 11.0%	0.838
Correct knowledge of disposal of used menstrual materials	48 (25%)	58 (30%)	+5.2	>0.05	60 (31%)	65 (34%)	+2.6	0.577	3.6%	−5.7% to 13.0%	0.444
Correct knowledge of changing menstrual materials as needed, at least every 6 hours	173 (90%)	163 (85%)	−5.2	>0.05	165 (86%)	165 (86%)	0.0	1.000	1.0%	−6.0% to 8.1%	0.772

### 3.3 Changes in attitudes toward menstruation

Attitudinal outcomes measured using the MAQ are presented in Table 4. At baseline, participants in both groups displayed moderately conservative or negative perceptions, viewing menstruation as debilitating and bothersome. Post-intervention, the intervention group showed a marked attitudinal shift toward normalization and empowerment, while the control group not significantly improved.

**Table 4:** Attitudes toward menstruation among participants (MAQ subscales).

MAQ subscale	Control baseline		Control endline		Intervention baseline		Intervention endline		Endline mean difference (Intervention − Control)	95% CI	Between-group p-value	Cohen’s d
	Mean ± SD	Median (IQR)	Mean ± SD	Median (IQR	Mean ± SD	Median (IQR	Mean ± SD	Median (IQR				
Menstruation as a debilitating event	4.7 ± 1.1	4.8 (4.0–5.5)	4.8 ± 1.0	4.8 (4.1–5.5)	4.6 ± 1.0	4.6 (3.9–5.3)	5.2 ± 1.1	5.2 (4.5–6.0)	0.40	0.19 to 0.61	<0.001	0.52
Menstruation as a bothersome event	4.9 ± 1.2	5.0 (4.1–5.8)	5.1 ± 1.2	5.2 (4.3–6.0)	4.3 ± 1.1	4.3 (3.5–5.1)	5.0 ± 1.0	5.0 (4.3–5.7)	−0.10	−0.32 to 0.12	0.376	0.44
Menstruation as a natural event	4.8 ± 1.1	4.8 (4.0–5.6)	5.1 ± 1.0	5.1 (4.4–5.8)	4.8 ± 1.0	4.8 (4.1–5.5)	5.9 ± 1.1	6.0 (5.2–6.7)	0.80	0.59 to 1.01	<0.001	0.68
Anticipation and prediction of menstruation†	4.5 ± 1.3	4.5 (3.6–5.4)	4.8 ± 1.2	4.8 (4.0–5.6)	4.4 ± 1.2	4.4 (3.6–5.2)	6.0 ± 1.1	6.1 (5.3–6.8)	1.20	0.97 to 1.43	<0.001	0.71
Denial of the effects of menstruation‡	4.4 ± 1.0	4.4 (3.7–5.1)	4.5 ± 1.0	4.5 (3.8–5.2)	4.2 ± 1.1	4.2 (3.4–5.0)	4.8 ± 1.0	4.8 (4.1–5.5)	0.30	0.10 to 0.50	0.003	0.36

In the intervention group, the mean score for the “menstruation as a natural event” subscale increased from 4.8 to 5.9, and the “anticipation and prediction” domain increased from 4.4 to 6.0. At endline, the intervention group scored significantly higher than the control group on the “menstruation as a natural event” domain (mean difference: 0.80; 95% CI: 0.59 to 1.01; p < 0.001), the “anticipation and prediction” domain (mean difference: 1.20; 95% CI: 0.97 to 1.43; p < 0.001), the “menstruation as a debilitating event” domain (mean difference: 0.40; 95% CI: 0.19 to 0.61; p < 0.001), and the “denial of effect” domain (mean difference: 0.30; 95% CI: 0.10 to 0.50; p = 0.003). The observed effect sizes indicate meaningful educational impact. In particular, the effect size for the “menstruation as a natural event” domain (Cohen’s d = 0.68) represents a moderate effect, while the “anticipation and prediction” domain (Cohen’s d = 0.71) approaches a large effect according to conventional thresholds. These findings suggest that the intervention contributed to more positive and less stigmatized menstrual attitudes among nursing students. In the Table 4, MAQ items were rated on a 7-point Likert scale ranging from 1 (strongly disagree) to 7 (strongly agree); therefore, the potential mean subscale score range was 1–7. The MAQ subscales represent conceptually distinct dimensions of menstrual attitudes, and higher scores should be interpreted according to the meaning of each subscale rather than uniformly as indicating a more positive attitude. Between-group p-values represent endline comparisons using independent-samples t-tests. Cohen’s d represents the standardized magnitude of baseline-to-endline change in the intervention group. †Anticipation and prediction of menstruation reflects awareness and perceived ability to anticipate menstruation based on physical, emotional, or other menstrual-cycle-related signs. ‡Denial of the effects of menstruation reflects a tendency to minimize or dismiss perceived physiological or psychological effects associated with menstruation. CI = confidence interval; IQR = interquartile range; MAQ = Menstrual Attitude Questionnaire; SD = standard deviation.

### 3.4 Changes in menstrual hygiene practices

Table 5 presents changes in selected MHH practices in the intervention and control groups. Several practices increased from baseline to endline in both groups, although the magnitude of change differed between groups. Reported consistent use of disposable sanitary pads increased from 31% to 88% in the intervention group and from 48% to 58% in the control group. At endline, disposable-pad use was 30.2 percentage points higher in the intervention group than in the control group (95% CI: 21.8 to 38.6; p < 0.001). Sample menstrual products were provided during the intervention demonstration and return after demonstration. The proportion reporting changing menstrual materials at least three times per day increased from 35% to 65% in the intervention group and from 26% to 39% in the control group, corresponding to an endline difference of 26.0 percentage points (95% CI: 16.4 to 35.7; p < 0.001). This measure is interpreted as a descriptive menstrual-management indicator rather than a universally appropriate hygiene threshold because changing or emptying frequency depends on menstrual flow and menstrual product type. Handwashing before changing menstrual materials increased from 45% to 76% in the intervention group and from 50% to 55% in the control group, with an endline difference of 20.8 percentage points (95% CI: 11.6 to 30.1; p < 0.001). Reported use of soap during genital washing increased from 29% to 47% in the intervention group and from 29% to 36% in the control group (endline difference: 10.9 percentage points; 95% CI: 1.1 to 20.7; p = 0.030). Wrapping used menstrual materials before disposal increased from 63% to 89% in the intervention group and from 63% to 70% in the control group (endline difference: 19.3 percentage points; 95% CI: 11.4 to 27.1; p < 0.001). Sun-drying reusable cloths increased from 50% to 55% in the intervention group while remaining at 43% in the control group (endline difference: 12.0 percentage points; 95% CI: 2.1 to 21.9; p = 0.019). Reported ironing of reusable menstrual materials increased from 23% to 78% in the intervention group and from 23% to 53% in the control group (endline difference: 25.0 percentage points; 95% CI: 15.8 to 34.2; p < 0.001). In the Table 5, Values are presented as n (%) of participants providing the prespecified reference/correct response. Absolute change is expressed as the percentage-point (pp) difference between baseline and endline within each group. Within-group changes were assessed separately for the intervention and control groups using McNemar’s test for paired binary responses. Between-group comparisons at endline were assessed using chi-square tests, with absolute differences in proportions and 95% confidence intervals (CI) reported. †Disposable sanitary pad use is a product-use indicator rather than an independent measure of intervention effectiveness. ‡Changing menstrual materials ≥3 times/day is a descriptive menstrual-management indicator rather than a universally appropriate hygiene threshold because appropriate changing or emptying frequency varies according to menstrual flow and product type. §Sun-drying and ironing apply specifically to reusable menstrual materials and should be interpreted in the context of reusable-material use.

**Table 5:** Menstrual hygiene practices of study participants.

Practice indicator	Control baselinen (%)	Control endlinen (%)	Control change (pp)	Assumed controlMcNemar p	Intervention baselinen (%)	Intervention endlinen (%)	Intervention change(pp)	InterventionMcNemar p	Endline difference(I − C)	95% CI	Between-groupp-value
Consistent use of disposable sanitary pads†	92 (48%)	111 (58%)	+9.9	0.012	60 (31%)	169 (88%)	+56.8	<0.001	30.2%	21.8% to 38.6%	<0.001
Changed menstrual materials ≥3 times/day‡	50 (26%)	75 (39%)	+13.0	0.003	67 (35%)	125 (65%)	+30.2	<0.001	26.0%	16.4% to 35.7%	<0.001
Handwashing before changing materials	96 (50%)	106 (55%)	+5.2	0.143	86 (45%)	146 (76%)	+31.3	<0.001	20.8%	11.6% to 30.1%	<0.001
Use of soap during genital washing	56 (29%)	69 (36%)	+6.8	0.048	56 (29%)	90 (47%)	+17.7	<0.001	10.9%	1.1% to 20.7%	0.030
Wrapped materials before disposal	121 (63%)	134 (70%)	+6.8	0.039	121 (63%)	171 (89%)	+26.0	<0.001	19.3%	11.4% to 27.1%	<0.001
Sun-dried reusable cloths§	83 (43%)	83 (43%)	0.0	1.000	96 (50%)	106 (55%)	+5.2	0.020	12.0%	2.1% to 21.9%	0.019
Ironed reusable materials§	44 (23%)	102 (53%)	+30.2	<0.001	44 (23%)	150 (78%)	+55.2	<0.001	25.0%	15.8% to 34.2%	<0.001

## 4. Discussion

This quasi-experimental study evaluated changes in MHH related KAP associated with a structured PE intervention among nursing and midwifery students in Bangladesh. The PE intervention was associated with significant improvements across multiple variables within the knowledge, attitude, and practice domains, although the magnitude and statistical significance of the observed differences varied across individual indicators. These findings suggest that short-term, interactive educational approaches may contribute to improvements in selected aspects of menstrual health knowledge, attitudes, and practices among health science students [37]. Importantly, the intervention’s participatory design, adapted from WaterAid’s Menstrual Hygiene Matters framework [9,33], incorporated interactive learning, discussion, and peer engagement to address menstrual KAP. Although the single-institution quasi-experimental design limits causal inference and broader generalizability, the observed findings suggest that PE education may be a useful approach for strengthening selected MHH-related competencies among nursing and midwifery students and supporting their future roles in menstrual health education and care.

### 4.1 Enhancing menstrual knowledge through participatory learning

The most striking outcome of this study was the substantial increase in menstrual knowledge following the one-month intervention. Students’ understanding of the menstrual cycle, physiological mechanisms, and appropriate hygiene practices improved dramatically; for instance, recognition of the average 28-day cycle rose from 8% to 78%, and correct identification of the uterine lining as the source of menstrual blood increased from 35% to 65%. These gains underscore the power of participatory and interactive pedagogy in health education. Comparable improvements have been reported in interventions conducted among adolescents in Iran [18] and secondary students in Nigeria [26], yet our study adds new evidence that such approaches are equally effective among nursing students. Despite their clinical background, many participants initially held misconceptions and cultural myths—revealing that professional education alone does not guarantee menstrual literacy. The observed cognitive shift aligns with Bloom’s taxonomy of learning, which posits that comprehension and knowledge acquisition are prerequisites for behavioral change [27,30]. The intervention succeeded in converting fragmented biomedical knowledge into integrated, gender-sensitive understanding, a crucial first step toward developing competent and empathetic future healthcare providers. However, improvements were not uniform across all knowledge domains. Some areas, such as uterine physiology, disposal practices, and recommended changing frequency, showed only limited improvement. These topics may require more repeated, practical, and context-specific reinforcement than a short four-session intervention can provide. In particular, concepts related to internal reproductive physiology may be difficult to retain without visual, anatomical, or simulation-based teaching, while disposal practices are strongly shaped by hostel infrastructure, privacy, and availability of appropriate waste-management facilities. Therefore, limited improvement in these domains may reflect both cognitive complexity and environmental constraints rather than lack of interest among students.

### 4.2 Changes in attitudes: From silence to empowerment

Beyond knowledge, participants showed positive changes in selected dimensions of menstrual attitudes following the intervention. Significant between-group differences were observed in three of the five MAQ domains at endline, including a higher score for perceiving menstruation as a natural event. This finding suggests that participatory education may contribute to greater recognition of menstruation as a normal physiological process. This positive attitudinal shift reflects the affective learning process described by Krathwohl, Bloom, and Masia [29], wherein emotional engagement and reflection enable learners to re-evaluate internalized beliefs [38]. The open-discussion and peer-sharing components of the intervention helped students break the silence surrounding menstruation, normalize discussion of bodily functions, and develop empathy toward others who face menstrual challenges. Such findings are consistent with prior studies in Ethiopia [39] and India [40], where participatory menstrual education reduced embarrassment and improved body image. However, changes were not consistent across all dimensions of menstrual attitudes. Two of the five MAQ domains did not show statistically significant between-group differences at endline. Several factors may explain this finding. Menstrual attitudes are multidimensional, and perceptions such as inconvenience or bothersomeness may be influenced not only by knowledge but also by menstrual pain, bleeding, personal experiences, social norms, and environmental conditions that a short educational intervention may be less able to modify. In addition, participants were nursing and midwifery students with prior health-related education, which may have limited the scope for change in some attitude dimensions. For nursing and midwifery students’ future advocates of women’s health these attitudinal improvements are particularly important. They not only influence personal comfort but also shape professional behavior when providing menstrual health counseling to patients. Ultimately, this shift from secrecy to empowerment suggests that affective learning can bridge knowledge and practice, contribute to greater openness in discussing menstruation as an aspect of health.

### 4.3 Changes in menstrual management practices

The PE intervention was associated with changes in several menstrual management practices. After the intervention, sanitary-pad use increased from 31% to 88%, frequency of changing materials at least three times per day rose from 35% to 65%, and regular hand-washing before and after changing materials reached 76% and 89%, respectively. Such changes may reflect not only acquired skills but also greater capability and readiness to adopt health-protective menstrual practices [28]. Previous WASH-linked menstrual interventions in Nepal [10] and Kenya [4] similarly demonstrated that behavioral improvement is sustained when educational content integrates practical demonstration, environmental awareness, and peer reinforcement. However, the substantial increase in disposable sanitary-pad use should not be attributed solely to the educational content of the intervention. Sample menstrual products were provided as part of the demonstration activities, and increased access to these products may have contributed to the observed change in product use. In addition, the educational content itself may have influenced participants’ product preferences or reporting of product use. Therefore, the observed increase in disposable-pad use may reflect a combination of education, product exposure, and access rather than a change in knowledge or attitudes alone. Notably, our participants also improved in environmentally safe practices such as sun-drying, and ironing of reusable materials. These changes are essential in the Bangladeshi context, where hostel living conditions often limit access to safe disposal and private drying areas. Thus, the findings were consistent with the proposed behavioral pathway of learning, suggesting that cognitive gains and positive attitudes, supported through modeling and feedback, may contribute to improved menstrual hygiene practices [32]. By linking menstrual education with practical skill-building, the program bridged the gap between “knowing” and “doing.”

### 4.4 Positioning nursing students as menstrual health advocates

A distinctive contribution of this study is its focus on nursing students rather than adolescents or adult women in general. As future frontline health professionals, nurses are uniquely positioned to influence public discourse and promote menstrual equity [20]. However, research indicates that even in professional settings, menstrual stigma persists and can undermine clinical empathy [41,42]. The observed changes in selected menstrual health KAP may have relevance for nursing and midwifery education because these students may encounter menstrual health concerns in their future clinical and community roles. However, the study did not directly assess students’ confidence in discussing menstruation, readiness to provide menstrual health counseling, or their subsequent professional behavior. Therefore, conclusions regarding these competencies cannot be drawn from the present findings. Nevertheless, incorporating structured menstrual health education into nursing and midwifery curricula may provide opportunities to strengthen students’ understanding of menstruation and prepare them to address menstrual health more appropriately in future practice. Further research should examine whether such educational interventions improve communication skills, counseling confidence, clinical competencies, and the provision of respectful menstrual health care.

### 4.5 Integration of menstrual health into nursing education and policy

The findings provide preliminary, context-specific evidence that participatory menstrual health education could be considered for further testing within nursing and midwifery education in Bangladesh. Before any national curriculum integration is considered, similar PE intervention modules should be evaluated across multiple nursing and midwifery institutions representing different geographic, socio-cultural, and administrative contexts. Integrating menstrual health into nursing education would advance several Sustainable Development Goals- SDG 3 (Good Health), SDG 4 (Quality Education), SDG 5 (Gender Equality), and SDG 6 (Clean Water and Sanitation) [6]. Curricular integration must also be accompanied by infrastructural improvements, such as accessible WASH facilities, private changing rooms, and adequate disposal mechanisms, which reinforce learned behaviors [3,43]. Similar policy shifts have been successful in India and Nepal, where WaterAid-supported programs established menstrual-friendly schools and training institutions [9,33]. Institutionalizing menstrual education in nursing can ensure that its future health workforce embodies the principles of equity, empathy, and evidence-based practice.

### 4.6 Comparison with global evidence and theoretical implications

The medium-to-large effect sizes observed in this study (0.68–0.82) align closely with findings from global meta-analyses, which report standardized mean differences between 0.5 and 0.8 for menstrual-education interventions [8]. This confirms the robustness of participatory educational approaches across cultural contexts. However, our findings extend the literature by suggesting the potential relevance of these approaches to tertiary health education, a context that has received comparatively limited attention.

Theoretically, this study supports the integration of social cognitive theory and affective learning frameworks in designing menstrual-health programs. The cognitive improvements observed mirror Bloom’s learning hierarchy, while attitudinal and behavioral changes reflect self-efficacy enhancement and the affective domain [28,29]. Together, these findings contribute to an emerging model of menstrual health promotion in higher education, where skill, emotion, and environment intersect to produce sustainable change.

### 4.7 Limitations

This study has several limitations. First, it was conducted at a single government nursing institution, which may limit the generalizability of the findings to nursing and midwifery students in other regions and institutions in Bangladesh. Only female students were included; therefore, the findings may not apply to male students, who may have different menstrual health knowledge and attitudes but may still provide menstrual health-related care and education in their future professional roles. Pregnant students were also excluded, which may have reduced the representation of some married or older students. Second, the quasi-experimental design and non-random allocation of existing class sections limit causal inference, and residual or unmeasured differences between groups cannot be excluded. Because intervention and control participants studied at the same institution and often shared academic and residential environments, informal information exchange may also have contributed to changes in the control group and reduced between-group differences. Third, approximately one-quarter of participants did not live-in student hostels. Although the intervention addressed menstrual health issues relevant across residential settings, components related to shared WASH facilities, privacy, disposal, and drying of reusable materials may have differed in relevance for hostel and non-hostel residents. The study was not designed to examine whether residential setting modified intervention outcomes. Fourth, KAP were self-reported and may have been affected by recall and social desirability bias. Some practice outcomes may also have been influenced by factors other than education. Sample menstrual products were demonstrated during intervention activities, and product access or exposure may have influenced subsequent product use. Moreover, appropriate changing frequency varies with menstrual flow and product type. As product-specific adjusted analyses were not conducted, differences related to product choice cannot be excluded. Fifth, only one post-intervention assessment was conducted, at six months. Without immediate or intermediate assessments, the trajectory of change and whether initial changes were maintained, diminished, or increased over time cannot be determined. The findings therefore do not establish the long-term sustainability of the observed differences. Sixth, the intervention focused primarily on students’ personal menstrual health knowledge, attitudes, and management practices and did not comprehensively address the clinical assessment and management of menstrual disorders. Topics such as prolonged or heavy menstrual bleeding, anemia, and potential underlying conditions such as bleeding disorders were not covered in detail. Future interventions should incorporate these clinical components to better prepare nursing and midwifery students to recognize menstrual health problems and provide appropriate counseling, care, and referral. The study also did not assess whether changes in KAP translated into clinical competencies, counseling skills, patient-care behaviors, or subsequent professional practice.

Despite these limitations, the study has several strengths, including its focus on an under-researched population, use of a theory-informed participatory educational approach, inclusion of a comparison group, and assessment of outcomes six months after the intervention. Future multi-site studies should use stronger designs, include students of different genders, incorporate repeated follow-up assessments, and examine the influence of residential setting and menstrual product type. Further research should also determine whether changes in personal menstrual health knowledge and practices are sustained over time and translate into clinically relevant competencies and professional practice.

### 4.8 Future directions

Future interventions should extend beyond personal menstrual health literacy to strengthen the clinical competencies of nursing and midwifery students in menstrual health assessment and care. This is particularly important because heavy menstrual bleeding can adversely affect health and well-being and has been associated with adverse outcomes among women in South Asia, including Bangladesh [44]. Future educational interventions could therefore incorporate recognition of abnormal menstrual bleeding and related danger signs, assessment of menstrual blood loss based on menstrual product use, identification of associated outcomes such as anemia, and recognition of potential underlying conditions, including uterine fibroids and bleeding disorders. Training could also introduce evidence-based management, counseling, and appropriate referral pathways for heavy menstrual bleeding, considering available medical and surgical treatment options [45]. Incorporating these clinical components may better prepare nursing and midwifery students to identify menstrual health problems and provide appropriate patient-centered support within their future professional roles.

## 5. Conclusion

This single-site quasi-experimental study found that a short PE intervention was associated with improvements in selected MHH-related KAP among nursing and midwifery students. The findings suggest that participatory menstrual health education may be a useful approach within nursing education; however, the non-randomized design and single-institution setting limit causal inference and generalizability. Larger, multi-site studies with stronger designs and repeated follow-up assessments are needed to confirm these findings and determine whether observed changes are sustained and translate into clinical practice.

## Supporting information

S1 File Questionnaire used to assess menstrual health and hygiene knowledge, attitudes, and practices. (DOCX)

## Abbreviations

BDT: Bangladeshi Taka
BNMC: Bangladesh Nursing and Midwifery Council
DGNM: Directorate General of Nursing and Midwifery
KAP: Knowledge, Attitude, and Practice
LMICs: Low- and Middle-Income Countries
MAQ: Menstrual Attitude Questionnaire
MHH: Menstrual Health and Hygiene
MHM: Menstrual Hygiene Management
MPQ: Menstrual Practice Questionnaire
PE: Participatory Educational
RNC: Rajshahi Nursing College
SDGs: Sustainable Development Goals
JMP Pro: Statistical software
UNICEF: United Nations Children's Fund
WASH: Water, Sanitation and Hygiene
WHO: World Health Organization.
