# Supplementary material for: Effectiveness of a participatory educational intervention on menstrual health and hygiene knowledge, attitudes, and practices among nursing and midwifery students in Bangladesh
Source: PLoS One. 2026 Nov 10;21(11):e0362603. doi: 10.1371/journal.pone.0362603 (PMC13750551; doi:10.1371/journal.pone.0362603)
Supplement: Supplementary file 1 [file pone.0362603.s002.pdf]

<table>
<tr><th colspan="2">Effectiveness of a Participatory Educational Intervention on Menstrual Health and Hygiene Knowledge, Attitudes, and Practices Among Nursing Students in Bangladesh</th></tr>
<tr><th colspan="2">Reviewer Comments</th></tr>
<tr><td>Overall impression</td><td>Overall, while this study includes nursing and midwifery students and discusses the potential implications of the intervention on their future clinical practice, the PE intervention appears primarily focused on students' own personal menstruation knowledge and practices, rather than preparing them as clinicians to assess menstruation clinically and recognize abnormal menstrual bleeding in patients. This includes danger signs/symptoms, how to estimate blood loss from menstrual product use, associated outcomes such as anemia, and underlying pathology (e.g., fibroids, bleeding disorders) that can contribute to heavy menstrual flow. As described, the intervention does not appear to address these clinically oriented topics, which is a gap, especially as the authors suggest that this intervention could inform potential changes to nursing curricula. Nursing and midwifery students are uniquely positioned to have these conversations with patients, and heavy menstrual bleeding is a recognized public health concern in Bangladesh (Charles et al., 2025, Am J Obstet Gynecol, DOI: 10.1016/j.ajog.2025.06.018). The authors may wish to consider incorporating content on clinical recognition and evidence-based management of heavy menstrual bleeding (e.g., Cochrane review on HMB treatment options), or, if this was intentionally outside the scope of the current intervention, please consider briefly noting this as a direction for future work in the discussion/limitations section or section 4.5.

Additionally, throughout the manuscript, several statements draw stronger causal or evaluative conclusions than the study design and data appear to support (e.g., use of terms like "transformation," "validated," and claims of improvement across all measured domains). Given the quasi-experimental, single-site, single-post-test design, I'd encourage the authors to adopt more tempered language throughout and ensure that all claims are clearly grounded in the reported results. Specific instances are noted in the line-by-line comments below.</td></tr>
<tr><th colspan="2">Manuscript</th></tr>
<tr><td>Abstract</td><td>• Suggest mentioning midwifery students, in addition to nursing, at least once in your title or abstract so that this inclusion criterion is clear.
Methods:
• Suggest reporting the post-evaluation timepoint as '6 months post-intervention' rather than calendar dates, as the current phrasing is difficult to follow (e.g. the intervention occurred over 4 weeks, yet the total study duration was 6 months).
• Suggest defining the WASH abbreviation for clarity, even though it may be a familiar term to most readers in this field.

Results:</td></tr>
</table>

	• The reported mean values do not have much meaning on their own as there are no descriptors of the scores or a possible range reported. Suggest adding some context or refining so results are more meaningful.<br>• On line 48, please amend to state "average menstrual cycle length"<br>• The phrase 'attitudes toward menstruation in anticipation and prediction of menstruation' is unclear, as it's not evident what construct is being measured (e.g., attitudes about predictability of one's cycle? anticipatory anxiety?). Suggest refining this wording for clarity<br><br>Conclusion:<br>• "*hereafter referred to as the PE intervention*" is not needed, as you do not use the PE abbreviation again in the abstract. You also previously defined this term in the methods section.<br>• In addition to the single-site limitation already noted, the study also had only one post-test evaluation, so the sustainability of any effects remains unclear (even though the evaluation occurred at 6 months, as this could be significantly lower than if testing was done immediately post). This should be amended or acknowledged in the abstract.
**INTRODUCTION: general comments**	Overall, the introduction is lengthy given the amount of background needed to orient the reader, and some details may fit better elsewhere: for example, study setting information (starting line 137) could move to the methods section, and intervention design details (page 7) could also move to methods or be removed altogether to avoid repetition. Similarly, content such as limitations related to the single-site design may be better suited to the discussion, and additional background detail could be moved to a supplement if not essential to the core narrative. Suggest refining and aiming for a more concise introduction, as public health manuscripts often favor this.
**Lines 111 -112**	• Although the meaning becomes clearer later in the manuscript, the term 'hostel settings' may not be familiar to all readers at first use. Suggest including a definition or refining the sentence for clarity.<br>• Do you have references demonstrating this purported cultural silence broadly across academic settings? If not, suggest narrowing the claim's scope, for example, specifying that it refers to academic and hostel settings in Bangladesh, and/or to the nursing field specifically, rather than academic settings in general.
**Lines 111-113**	Suggest adding 'and their own menstrual health', as you note on line 120
**Line 123**	The referent of "both groups" is unclear, please clarify which two groups this refers to (e.g., nursing and midwifery students, intervention and control groups, or another pairing).
**Lines 124-125**	The claim that nursing and midwifery students are a "crucial but under-examined population in menstrual health research" is a strong empirical statement that warrants a supporting citation (e.g., a systematic review or bibliometric analysis demonstrating this gap). As written, it's unclear what evidence supports this characterization. Suggest either citing a source

	that substantiates this gap, or narrowing the claim to a more specific and verifiable scope, for example, "under-examined in the context of a specific outcome/intervention type/geographic setting."
**Lines 130-133**	Suggest including a reference to support the claimed "multiplier effect" of MHH literacy
**Lines 165-167**	Please provide a reference to support the claim that group dialogue and role-play activities reduce embarrassment. This may not hold universally as for some students, these activities could increase discomfort, particularly given the stigmatized nature of the topic. Consider citing evidence or acknowledging this as a possible limitation/individual variation.
**Study Design**	The manuscript describes the overall design as quasi-experimental (i.e., non-randomized), which appears to refer to how students were assigned to intervention versus control groups. However, the text also states that randomization occurred by study year, with Figure 2 reporting stratified random sampling. This is a potentially important distinction that isn't currently clear: stratified random sampling of participants within each study-year stratum (which affects who was measured) is different from random assignment of groups to intervention or control (which affects internal validity/causal inference). As currently written and depicted in Figure 2, it reads as though randomization may have addressed the quasi-experimental limitation, when it may only pertain to participant selection rather than group assignment. Please clarify explicitly: (1) how students/class sections were assigned to intervention vs. control (and whether this was random or not), and (2) how the stratified random sampling by study year fits into that process. This distinction has direct implications for how causal claims in the results/discussion should be framed.
**Line 186**	Strongly suggest refining the statement that "meaningful causal inference" can be drawn from this pre/post design. While the addition of a control group strengthens the design relative to a single-group pre/post study, this was not an RCT, nor a controlled interrupted time series with multiple evaluations, and the study was conducted at a single hostel institution, which raises contamination and confounding risks discussed elsewhere in this review. In addition, knowledge, attitudes, and practices are self-reported and may be subject to social desirability bias, particularly among students who may feel pressure to report what they perceive is expected of them post-intervention.
**Lines 213-215 and 232-235**	These statements would be more appropriate under limitations in the discussion
**Lines 216-217**	The reported ages of nursing students are a study result (i.e., a characteristic of the enrolled sample) and would typically be reported in the results section, not methods, unless a specific age range was a pre-specified eligibility/inclusion criterion, in which case that criterion (not the sample's actual observed ages) belongs in methods.
**Line 240**	The response rate of 96.7% is a study result and would be more appropriate in the results section rather than methods.
**Section 2.3**	Please include a reference for G*Power as you have done for JMP Pro on line 352

**Line 337**	It is unclear why interviewer bias is listed as a concern, given that self-administered surveys were used. Interviewer bias typically applies to interviewer-administered data collection. Please clarify whether this refers to a different aspect of data collection (e.g., bias introduced during survey distribution or clarification of questions) or remove/revise if not applicable.
**Lines 349-350**	Please clarify why daily questionnaire checks for completeness were conducted, given that the study design included only single pre- and post-test assessments. If data collection occurred over multiple days within each timepoint (e.g., surveys administered across a data collection window rather than a single day), please specify this in the text.
**Line 353**	Please provide more explanation/rationale for removing outliers, specifically, what statistical criteria or threshold defined an "outlier" (e.g., ±3 SD, IQR method), how many data points were removed, and whether this decision was pre-specified or made post hoc after seeing the data. Post hoc outlier removal without a clear, pre-specified rule can introduce bias into the results.
**Section 3.1**	Suggest reducing the text here to focus on the most important participant characteristics and then refer the reader to Table 1 rather than describing all demographics in prose.
**Table 1**	Approximately one-quarter of the sample did not reside in a hostel, yet the methods describe the intervention as adapted specifically for students in a hostel environment.<br>• Were any sub-analyses conducted comparing hostel vs. non-hostel residents, or is this discrepancy addressed elsewhere? If not, please consider discussing whether/how non-hostel residents' inclusion affects the intervention's relevance or the interpretation of results for this subgroup.<br>• Some variables do not sum to the reported total (n=192). Based on the questionnaires in the supplement, this discrepancy may reflect unreported categories (e.g., "other" religion) and/or missing data. Please confirm the reported n for each variable and/or provide additional detail in the supplement (e.g., missing data counts per variable) for clarity and transparency.
**Table 2**	• Strongly suggest relabeling the variable "*correct age at menarche (13 years)*." Menarche onset varies normally across individuals and populations, and typical onset has also changed over time (Papadimitriou, 2016, J Pediatr Adolesc Gynecol, DOI: 10.1016/j.jpag.2015.12.002). There is nothing inherently "correct" about 13 years specifically and framing it this way risks reinforcing the misconception among nurses, girls, and women that menarche outside this age is abnormal or "incorrect." Consider relabeling as something like "knowledge of typical/average age range at menarche" and clarifying in the text what age or range was used as the reference answer and why.<br>• Please strongly consider including a column showing the within-group McNemar p-value for the control group as well. This is particularly important given that, for a couple of variables, the

	control group showed a greater absolute change pre/post than the intervention group. Reporting the McNemar result only for the intervention group is a form of selective or outcome reporting bias, and it obscures the observed changes in the control group. As currently presented, this could be misinterpreted to imply that control-group changes were not significant, when the between-group p-values show that only 2 variables remained significant overall. Please either include the within-group McNemar column for both groups, or remove it for the intervention group alone.
**Table 3**	• Consider adding a footnote defining the potential range of scores (1-7) to improve clarity<br>• Consider reporting median and IQR in addition to means, given that these are ordinal (1-7 scale) variables — median/IQR may better reflect central tendency and spread if distributions are skewed or show ceiling/floor effects. This could be included in the main table or supplement.<br>• Suggest relabeling or adding footnotes to clarify the "anticipation and prediction" and "denial of effect" variable labels as their meaning is not immediately clear from the label alone.
**Lines 473-479**	Increased product access, rather than the educational content itself, could plausibly explain some of the observed increases, since the intervention group was given sample menstrual products, and it's unclear whether these were retained after the demonstration. Additionally, if control group participants used different product types (e.g., menstrual cups) at different rates, the "changing materials ≥3 times/day" indicator may not be an equally valid practice marker across product types. Please clarify sample product retention and whether this indicator is appropriately interpreted across products. As product-type data appear to have been collected for each participant (based on supplement), consider adjusting for product type as a covariate in the analysis to account for this potential confound.
**Lines 499-503**	This statement is too strong, as some domains showed non-significant between-group changes, particularly among knowledge indicators. Suggest refining (e.g., "the PE intervention was associated with significant improvements across multiple variables within the knowledge, attitudes, and practice domains" rather than implying uniform change across all domains).
**Lines 503-508**	These statements seem a bit strong for a single institution pre/post study, please refine for accuracy
**Lines 535**	Again, this statement seems too strong for what the data in Table 3 support. Of the 5 variables assessed, only 3 showed significant between-group differences, and the effect size for these was medium, not large or "profound." Language such as "associated with" would be a more accurate descriptor than "transformed," particularly given the single-center, pre/post design.
**Section 4.2**	It would also be informative to discuss possible reasons why 2 of the 5 variables did not reach statistical significance, rather than focusing discussion solely on the significant findings.
**Section 4.3**	As noted above, please clarify whether these changes could be at least partially attributed to the intervention group's access to distributed

	supplies, which may have made certain practices more directly achievable regardless of any change in knowledge or attitudes.<br><br>Relatedly, based on the survey questions in the supplement, did product use differ within each group between pre- and post-test? This is worth considering even independent of physical product distribution: if the intervention content characterized disposable pads as a superior product, this messaging alone could have shifted participants' product choices or their self-reported responses, separate from any change in underlying knowledge or attitudes. Ideally, the analysis should adjust for product type as a covariate to help disentangle these effects from the intervention's informational content.<br><br>Lastly, the heading and text (lines 556-557) state that the observed changes demonstrate improved self-efficacy. However, self-efficacy is a distinct, well-defined construct, and while improvements in KAP may suggest enhanced capability or readiness to engage in the target behavior, they do not independently establish increased self-efficacy, which does not appear to have been directly measured in this study. The following references provide more information:<br>https://pubmed.ncbi.nlm.nih.gov/27280365/<br>https://pubmed.ncbi.nlm.nih.gov/10938914/
**Lines 561-562**	This statement indicates significant between-group improvement in sun-drying practices; however, this does not appear to be supported by the between-group results in Table 3. Please review and correct this discrepancy.
**Lines 564-567**	Please address whether other factors, such as the provision of materials, may have also contributed to these outcomes. Also, since there was only a single post-test measurement (albeit at a meaningful 6-month follow-up) it may be overly strong to state that the behavioral pathway for sustainable hygiene behaviors has been "validated." Consider softer language (e.g., "these findings provide preliminary support for..." or "suggest a potential pathway toward...") that better reflects the single-timepoint design.
**Limitations**	Suggest adding the following limitations:<br>• This study did not include any male nursing students, who may have different (or less) baseline knowledge and attitudes related to menstrual hygiene management (MHH), yet may still provide MHH-related care or education to patients in future clinical practice. This is worth noting as a limitation affecting the generalizability of findings to the full nursing/midwifery student population.<br>• As previously noted, the limitations section should also discuss the educational intervention's scope, specifically, its lack of coverage of clinically oriented content such as recognition of atypical menstrual health (e.g., prolonged bleeding, heavy menstrual bleeding, and associated risks such as anemia or bleeding disorders).

**Lines 573-582**	I could not identify any results or survey questions relating to students' confidence discussing menstruation or readiness to discuss it with future patients. Were these anecdotal reports rather than measured outcomes? If so, please clarify the source of this data. Also, suggest revising the phrases "this transformation is significant" and "building institutional educational capacity for gender-sensitive nursing education," as both interpretations appear to go beyond what was actually measured in this study.
**Fig 1**	Suggest revising this figure as:<br>1. The mechanisms depicted are unclear. For example, how do "human resources" reduce stigma, and how do "participant characteristics" lead to increased hygiene behavior? Passive resources, inherent participant traits, and environment alone likely do not bring about change on their own, which seems to be precisely why a participatory educational (PE) intervention was used, but this reasoning isn't reflected in the current framework.<br>2. The PE intervention is not clearly evident in this figure<br>3. Consider using "improved hygiene KAP" rather than "sustainable hygiene behaviour," or positioning KAP as an intermediate outcome rather than an endpoint. Additionally, "supporting institutional culture" is listed as an outcome but does not appear to have been measured in this study.<br><br>One option to address these issues would be to use an implementation science causal pathway diagram (Klasnja et al., 2024, Implement Res Pract, DOI: 10.1177/26334895241248851) to explicitly map the intervention, its theorized mechanism(s) of action, anticipated proximal and distal outcomes, necessary pre-conditions, and relevant moderators. This would clarify the causal logic connecting the PE intervention to hypothesized outcomes more precisely than the current figure.
**Fig 2**	Please see comments above under study design
**Supplement**	Please double check your supplemental tables as it appears some formatting may need fixing (e.g. duplicate numbering in the MAQ table)
