# Supplementary material for: Effectiveness of a participatory educational intervention on menstrual health and hygiene knowledge, attitudes, and practices among nursing and midwifery students in Bangladesh
Source: PLoS One. 2026 Nov 10;21(11):e0362603. doi: 10.1371/journal.pone.0362603 (PMC13750551; doi:10.1371/journal.pone.0362603)
Supplement: Supplementary file 2 [file pone.0362603.s001.docx]

i) **SOCIO-DEMOGRAPHIC CHARACTERISTICS**

| 1. | Current age | |  |
| --- | --- | --- | --- |
| 2. | How old were you when you started menstruating | |  |
| 3. | Category of studentship | Nursing |  |
|  |  | Midwifery |  |
| 4. | Name of Course | Diploma in Midwifery |  |
|  |  | B.Sc in Nursing (Post Basic) |  |
|  |  | B.Sc in Nursing (4 years) |  |
| 5. | Year of college | First year |  |
|  |  | Second year |  |
|  |  | Third year |  |
|  |  | Fourth year |  |
| 6. | Religion | Islam |  |
|  |  | Hinduism |  |
|  |  | Buddhism |  |
|  |  | Christian |  |
|  |  | Other |  |
| 7. | Fathers Education level | Graduate or above |  |
|  |  | Higher Secondary |  |
|  |  | Secondary |  |
|  |  | Primary |  |
|  |  | Illiterate |  |
| 8. | Mother’s Education level | Graduate or above |  |
|  |  | Higher Secondary |  |
|  |  | Secondary |  |
|  |  | Primary |  |
|  |  | Illiterate |  |
| 9. | Birthplace status | Urban |  |
|  |  | Rural |  |
| 10. | Resident status | Hostel |  |
|  |  | Own house |  |
| 11. | Monthly Family Income | Below 10000/- |  |
|  |  | 1000-20000/- |  |
|  |  | 20000-30000/- |  |
|  |  | More than 30000/- |  |
| 12. | Income Source | Father |  |
|  |  | Mother |  |
|  |  | Both |  |
|  |  | Others |  |
| 13. | Weight (kg) | |  |
| 14. | Height (cm) | |  |
| 15. | How many days did you miss the school/Class in the last menstrual period due to menstruation? | |  |

**KNOWLEDGE, ATTITUDE AND PRACTICE (KAP) QUESTIONNAIRE**

ii) **For Knowledge** (Alam *et al.,* 2022)
1. Do you know what is the average age of a girl’s first period/menarche?
   a. Year
   b. Don’t know
2. During menstruation, what is the reason or purpose for the monthly bleeding? **(multiple responses allowed here)**
a. To cleanse toxins from the body
b. To pass the old lining of the uterus from the body so a fresh lining can be formed
c. To indicate that there is a problem with the girl’s reproductive organs
d. To indicate that the girl is sick
e. Others
f. Don’t Know
3. Some girls and women have longer menstruation cycle, others have shorter menstruation cycle. On **average**, how often does a girl or woman typically get a period (in other words, how long is the entire cycle)?
a. ……….Days
b. Don’t know
4. Some girls and women have longer periods of bleeding, others have shorter periods of bleeding. On **average**, how long is each period of bleeding?
a. 1-3 days
b. 3-7 days
c. 7-10 days
d. More than 10 days
e. Don’t know
5. Could you please identify the name of the body part from the following picture? (Please show the photo to the respondent and tick whether she could mention following; If respondent can’t say any then tick on don’t know)
a.Ovaries
b. Fallopian tubes
c. Uterus
d. Vagina
e. Don’t know
6. Can you list at least three methods for reducing pain if a girl experiences any physical discomfort during her period? **(multiple responses allowed here)**
a. Pain medication
b. Exercise
c. Hot fomentation
d. Cold fomentation
e. Sleeping
f. Rest
g. Others
7. What is the proper way to wash and dry reusable sanitary products (cloths)? **(multiple responses allowed here)**
a. Wash with water
b. Wash with water and soap
c. Clothes are dried in such a way that they can dry completely
d. Others
8. What is the proper way to dispose of disposable sanitary products? **(multiple responses allowed here)**
a. Dispose in the waste bin
b. Wrapped and placed in a dustbin
c. Tossed into the pit of the latrine
d. Tossed in a canal
e. Incinerated
f. Thrown outside in the bush
g. Others
9. How often should sanitary products be changed?
a. As needed, according to blood flow, but AT LEAST every six hours
b. Every hour, regardless of blood flow
c. Once per day
d. As needed, but AT LEAST every 9 hours
e. Others
iii) **For Attitude: Menstrual Attitudes Questionnaire (MAQ) (** Brooks & Ruble 1980)

|  | **Variables** | **Strongly Disagree** |  |  |  |  |  | **Strongly Agree** |
| --- | --- | --- | --- | --- | --- | --- | --- | --- |
| **Menstruation as a debilitating event** | | **1** | **2** | **3** | **4** | **5** | **6** | **7** |
| 10. | A woman's performance in sports is not affected negatively by menstruation. |  |  |  |  |  |  |  |
| 11. | Women are more tired than usual when they are menstruating. |  |  |  |  |  |  |  |
| 12. | I expect extra consideration from my friends when I am menstruating. |  |  |  |  |  |  |  |
| 13. | Menstruation can adversely affect my performance in sports. |  |  |  |  |  |  |  |
| 14. | I feel as fit during menstruation as I do during any other time of the month. |  |  |  |  |  |  |  |
| 15. | I don't allow the fact that I'm menstruating to interfere with my usual activities. |  |  |  |  |  |  |  |
| 16. | Avoiding certain activities during menstruation is often very wise. |  |  |  |  |  |  |  |
| 17. | I am more easily upset during my premenstrual or menstrual periods than at other times of the month. |  |  |  |  |  |  |  |
| 18. | I don't believe my menstrual period affects how well I do on intellectual tasks. |  |  |  |  |  |  |  |
| 19. | I realize that I cannot expect as much of myself during menstruation compared to the rest of the month. |  |  |  |  |  |  |  |
| 20. | Women just have to accept the fact that they may not perform as well when they are menstruating. |  |  |  |  |  |  |  |
| **Menstruation as a bothersome event** | |  |  |  |  |  |  |  |
| 21. | Menstruation is something I just have to put up with. |  |  |  |  |  |  |  |
| 22. | In some ways I enjoy my menstrual periods. |  |  |  |  |  |  |  |
| 23. | Men have a real advantage in not having the monthly interruption of a menstrual period. |  |  |  |  |  |  |  |
| 24. | I hope it will be possible someday to get a menstrual period over within a few minutes. |  |  |  |  |  |  |  |
| 25. | The only thing menstruation is good for is to let me know I'm not pregnant. |  |  |  |  |  |  |  |
| 26. | Menstruation provides a way for me to keep in touch with my body. |  |  |  |  |  |  |  |
| **Menstruation as a natural event** | |  |  |  |  |  |  |  |
| 27. | Menstruation is a reoccurring affirmation of womanhood. |  |  |  |  |  |  |  |
| 28. | Menstruation allows women to be more aware of their bodies. |  |  |  |  |  |  |  |
| 29. | Menstruation provides a way for me to keep in touch with my body. |  |  |  |  |  |  |  |
| 30. | Menstruation is an obvious example of the rhythmicity which pervades all of life. |  |  |  |  |  |  |  |
| 31. | The recurrent monthly flow of menstruation if an external indication of a woman's general good health. |  |  |  |  |  |  |  |
| **Anticipation and prediction of the onset of menstruation** | |  |  |  |  |  |  |  |
| 32. | I can tell my period is approaching because of breast tenderness, backache, cramps, or other physical signs. |  |  |  |  |  |  |  |
| 33. | I have learned to anticipate my menstrual period by the mood changes which precede it. |  |  |  |  |  |  |  |
| 34. | My own moods are not influenced in any major way by the phase of my menstrual cycle. |  |  |  |  |  |  |  |
| 35. | I am more easily upset during my premenstrual or menstrual periods than at other times of the month. |  |  |  |  |  |  |  |
| 36. | Most women show a weight gain before or during menstruation. |  |  |  |  |  |  |  |
| **Denial of any effect of menstruation** | |  |  |  |  |  |  |  |
| 37. | Cramps are bothersome only if one pays attention to them. |  |  |  |  |  |  |  |
| 38. | A woman who attributes her irritability to her approaching period is neurotic. |  |  |  |  |  |  |  |
| 39. | I barely notice the physiological effects of my menstrual periods. |  |  |  |  |  |  |  |
| 40. | Women who complain of menstrual distress are just using that as an excuse. |  |  |  |  |  |  |  |
| 41. | Premenstrual tensionirritability is all in a woman's head. |  |  |  |  |  |  |  |
| 42. | Most women make too much of the minor physiological effects of menstruation. |  |  |  |  |  |  |  |

iv) **For Practice: Menstrual Practice Questionnaire (MPQ)** (Hennegan et al., 2022)

***Menstrual Material Use***

43. What were all the materials you used to catch/absorb your menstruation when you were at home during your last menstrual period? (select all that apply)

• Cloth/towel

• Disposable sanitary pad

• Reusable sanitary pad

• Toilet paper

• Cotton wool

• Mattress or foam

• Underwear alone

• Natural material (e.g., leaves, sand, grass)

• Period underwear

• Menstrual cup

• Tampon

• Other (specify)

44. What were all the materials you used to catch/absorb your menstruation when you were away from home [at school/at work] during your last menstrual period? (select all that apply)

• Cloth/towel

• Disposable sanitary pad

• Reusable sanitary pad

• Toilet paper

• Cotton wool

• Mattress or foam

• Underwear alone

• Natural material (e.g., leaves, sand, grass)

• Period underwear

• Menstrual cup

• Tampon

• Other (specify)

45. [Cloth users] Were your cloths bought to be used for menstruation or used for something else first? (select one)

• Bought to be used during menstruation

• Used for something else first (e.g., clothes, bedding)

• Don’t know

46. Did you wash and reuse any of your menstrual materials during your last menstrual period? (select one)

• No

• Yes

***Changing menstrual materials***

47. During your last menstrual period, how many times did you change your menstrual material on the heaviest day of your period? (day = 24 hours) (select one)

• 1 time (wear until the next day)

• 2 times (eg. morning and evening)

• 3 times (eg. morning, evening and once during day)

• 4 times (eg. morning, evening, and twice during day)

• More than 4 times

48. Where did you most often change your menstrual materials when you were at home during your menstrual last period? (select one)

• Latrine

• Bedroom

• Bathroom/washing space (separate from latrine)

• Outside/bush/field

• Other (specify)

49. How often did you change your menstrual materials when you were away from your home [at school/at work] during your last menstrual period? (e.g., school, market, working outside the home) (select one)

• Every day of my period

• Some days

• One day

• Never/no days

50. Where did you most often change your menstrual materials when you were away from your home [at school/at work] during your last menstrual period? (select one)

• Latrine

• A bathroom (separate from latrine)

• Another room at the location

• Outside/bush/field

• Other (specify)

***Handwashing***

51. Did you wash your hands before changing your menstrual materials during your last menstrual period? (select one)

• Never

• Sometimes

• Every time

52. Did you wash your hands after changing your menstrual materials during your last menstrual period? (select one)

• Never

• Sometimes

• Every time

***Genital washing***

53. How often did you wash your genitals during your last menstrual period? (select one)

• At the end of my period only

• Every 2-3 days

• Once per day

• Twice per day

• Three or more times per day

54. When you washed your genitals, did you use soap? (select one)

• Never

• Sometimes

• Every time

***Disposal of menstrual materials***

55. Where did you most often dispose of your used menstrual materials when you were at home during your last menstrual period? (select one)

• Into the latrine/toilet

• Burned

• Household rubbish (bin in latrine)

• Household rubbish (bin not in latrine)

• Taken to community rubbish

• Buried/bush/waterway

• Did not dispose of any materials (including reusables)

• Other (specify)

56. Where did you most often dispose of your used menstrual materials when you were away from your home [at school/at work] during your last menstrual period? (select one)

• Transported home to dispose or reuse

• Into the latrine/toilet

• Bin in the latrine/toilet

• Bin onsite but outside of the latrine/toilet

• Community rubbish (not onsite)

• Bush/buried/waterway

• Burned

• Other (specify)

57. When disposing of your used menstrual materials, did you usually wrap them in anything? (select one)

• No

• Yes, plastic bag, cover of pad

• Yes, toilet paper

• Yes, cloth

• Yes, other

***Storage of menstrual materials***

58. After your last menstrual period, did you keep [store] your menstrual materials? (includes leftover disposables or reusables) (select one)

• No

• Yes

59. Where did you store your menstrual materials after your last menstrual period? (select one)

• Cupboard, cabinet or drawer

• Under bed

• In the toilet/latrine room

• In a bathroom (not latrine/toilet)

• Other

60. Did you store your menstrual materials inside any wrapping, packaging or case? (select one)

• Wrapped in plastic or in plastic bag (including disposable pads in original packaging)

• Wrapped in fabric or in a fabric bag

• Wrapped in paper

• Cardboard box

• No wrapping or container

• Other

***Washing materials***

61. Did you soak your materials when washing them during your last menstrual period? How long did you usually soak them for?

[Time] 0 is did not soak

62. Where did you most often wash your menstrual materials during your last menstrual period? (select one)

• Shared bucket (or similar container)

• Own bucket (or similar container)

• Shower/bath

• Sink/basin (inside home)

• Sink/basin (outside home)

• Well, spring, waterway

• Other

63. Did you use soap or detergent to wash or soak your menstrual materials during your last menstrual period? (select one)

• Never

• Sometimes

• Every time

***Drying materials***

64. Where did you most often dry your menstrual materials during your last menstrual period? (select one)

• Outside (hanging)

• Outside (hidden)

• Inside (hanging)

• Inside (hidden)

• Other

65. When your menstrual materials were drying, did you usually cover them with anything? (select one)

• No

• Yes

66. When your menstrual materials were drying during your last menstrual period, were they in the sun? (select one)

• Never

• Sometimes

• All the time

67. During your last period, were your menstrual materials completely dry before you used them? (select one)

• Never

• Sometimes

• Every time

***Additional sterilisation practices***

68. [Fabric reusables] Did you use an iron on your menstrual materials before you reused them during your last menstrual period? (select one)

• Never

• Sometimes

• Every time

***Toilet/latrine practice during menstruation***

69. During your last menstrual period, when you were at home did you use the same location for urination as when you do not have your menstrual period? (select one)

• Never

• Sometimes

• All the time

70. During your menstrual last period, when you were at work did you use the same location for urination as when you do not have your menstrual period? (select one)

• Never

• Sometimes

• Every time
