# Supplementary material for: Effectiveness of a participatory educational intervention on menstrual health and hygiene knowledge, attitudes, and practices among nursing and midwifery students in Bangladesh
Source: PLoS One. 2026 Nov 10;21(11):e0362603. doi: 10.1371/journal.pone.0362603 (PMC13750551; doi:10.1371/journal.pone.0362603)
Supplement: Supplementary file 3 [file pone.0362603.s003.docx]

The manuscript is well-written in a structured format. However, please conside the minor suggestions:

1. In line 100, “menstruation is often treated as a secretive and impure phenomenon” the wording “secretive and impure” needs to be improved
2. In the method section, please clarify how 100% retention rate was achieved (line 343-345)
3. It is recommended to mention the IRB approval registration number and details.
4. Define PE (line 144)
5. Relying on t-test is an outdated approach. It is recommended to perform ANCOVA test.
