# Supplementary material for: Effectiveness of a participatory educational intervention on menstrual health and hygiene knowledge, attitudes, and practices among nursing and midwifery students in Bangladesh
Source: PLoS One. 2026 Nov 10;21(11):e0362603. doi: 10.1371/journal.pone.0362603 (PMC13750551; doi:10.1371/journal.pone.0362603)
Supplement: Supplementary file 4 [file pone.0362603.s005.docx]

**Response to the Academic Editor and Reviewers**

**Manuscript ID: PONE-D-26-40606**

*Effectiveness of a Participatory Educational Intervention on Menstrual Health and Hygiene Knowledge, Attitudes, and Practices Among Nursing Students in Bangladesh*

Dear Dr. Parker,

Thank you for the opportunity to revise our manuscript and for the thoughtful guidance from you and the reviewers. We have carefully revised the manuscript in response to the comments. In particular, we have strengthened methodological transparency, clarified participant sampling and non-randomized class-section allocation, moderated causal and evaluative language, improved reporting of effect estimates and confidence intervals, expanded the limitations, revised the figures and supporting information, and shortened the Introduction and Conclusion. Our point-by-point responses are provided below.

**Responses to Academic Editor and Journal Requirements**

**Academic Editor - overall guidance:** We carefully addressed the substantial comments from Reviewers 1 and 2 and moderated the strength of the conclusions throughout the manuscript. In accordance with your instruction, we did not add the ANOVA/ANCOVA analysis suggested by Reviewer 3. Instead, we retained the prespecified analyses and strengthened reporting of effect estimates, confidence intervals, baseline differences, and design limitations.

**1. PLOS ONE style requirements:** We reviewed the revised manuscript for PLOS ONE formatting and file-naming requirements and corrected the presentation accordingly.

**2. Inclusivity in Global Research questionnaire:** Completed. The PLOS questionnaire on inclusivity in global research has been completed and will be uploaded as Supporting Information with the revised submission.

**3. Information removed for blind review:** We restored the relevant author and institutional information in the revised manuscript because PLOS ONE peer review is not double-blinded.

**4. Funding Information and Financial Disclosure:** We reviewed and reconciled the funding information and financial disclosure so that the grant information is consistent across the manuscript and submission materials.

**5. Role of the funder:** We added the requested statement: 'The funders had no role in study design, data collection and analysis, decision to publish, or preparation of the manuscript.' This statement will also be included in the cover letter.

**6. Data Availability:** We prepared a de-identified/anonymized minimal dataset and associated metadata and deposited them in Zenodo. The Data Availability Statement has been updated accordingly: 'The anonymized dataset and associated metadata underlying the findings of this study are publicly available in the Zenodo repository at https://doi.org/10.5281/zenodo.23224070.'

**7. Ethics statement:** The ethics statement has been retained only in the Methods section, and the approving ethics/IRB body and approval/registration details are reported there.

**8. Figure copyright:** We replaced/revised the figure so that it is an original figure created by the authors and does not reproduce copyrighted third-party images. The revised figure is therefore suitable for publication under the journal's CC BY 4.0 license.

**Supporting Information captions:** We added captions for the Supporting Information files at the end of the manuscript and checked the corresponding in-text citations for consistency.

**Previously published works suggested by reviewers:** We reviewed the publications suggested by the reviewers and cited those that were relevant to the revised manuscript, including literature addressing heavy menstrual bleeding and related methodological/theoretical issues.

**Point-by-Point Responses to Reviewers**

We again thank the Academic Editor and reviewers for their constructive comments, which have substantially improved the clarity and rigor of the manuscript.

Sincerely,
The Authors

| **Reviewer comment** | **Author response** | **Line (L)** |
| --- | --- | --- |
| **Reviewer #1:** |  |  |
| Reviewer #1: Dear Authors, I hope this message finds you well.  Thank you for the opportunity to review this manuscript — menstrual hygiene management (MHH) knowledge, attitudes, and practices (KAP) among nursing and midwifery students in Bangladesh is an important and timely topic, and I appreciate the effort that has gone into this work.  In its current form, the manuscript requires revision in several areas, particularly with respect to length, methodological clarity, and the strength of causal and evaluative language used to describe the findings.  I have provided detailed comments throughout (see attached), and I want to acknowledge upfront that the volume of feedback may appear considerable. This reflects the complexity of the study design rather than a lack of merit in the work itself. I have tried, wherever possible, to explain the reasoning behind each comment and to offer concrete suggestions, with the intention of helping to strengthen the manuscript's rigor, clarity, and overall contribution. I hope the comments are received in that spirit. | We sincerely thank the reviewer for the careful, constructive, and detailed assessment of our manuscript. We have revised the manuscript extensively in response to these comments, with particular attention to methodological clarity, concise reporting, interpretation of the quasi-experimental design, and moderation of causal and evaluative language. | Throughout revised manuscript |
| **Overall impression**  Overall, while this study includes nursing and midwifery students and discusses the potential implications of the intervention on their future clinical practice, the PE intervention appears primarily focused on students' own personal menstruation knowledge and practices, rather than preparing them as clinicians to assess menstruation clinically and recognize abnormal menstrual bleeding in patients. This includes danger signs/symptoms, how to estimate blood loss from menstrual product use, associated outcomes such as anemia, and underlying pathology (e.g., fibroids, bleeding disorders) that can contribute to heavy menstrual flow. As described, the intervention does not appear to address these clinically oriented topics, which is a gap, especially as the authors suggest that this intervention could inform potential changes to nursing curricula. Nursing and midwifery students are uniquely positioned to have these conversations with patients, and heavy menstrual bleeding is a recognized public health concern in Bangladesh (Charles et al., 2025, Am J Obstet Gynecol, DOI: 10.1016/j.ajog.2025.06.018). The authors may wish to consider incorporating content on clinical recognition and evidence-based management of heavy menstrual bleeding (e.g., Cochrane review on HMB treatment options), or, if this was intentionally outside the scope of the current intervention, please consider briefly noting this as a direction for future work in the discussion/limitations section or section 4.5.  Additionally, throughout the manuscript, several statements draw stronger causal or evaluative conclusions than the study design and data appear to support (e.g., use of terms like "transformation," "validated," and claims of improvement across all measured domains). Given the quasi-experimental, single-site, single-post-test design, I'd encourage the authors to adopt more tempered language throughout and ensure that all claims are clearly grounded in the reported results. Specific instances are noted in the line-by-line comments below. | Thank you for this important overall assessment. We agree that the intervention primarily addressed students' personal menstrual health knowledge, attitudes, and management practices rather than clinical assessment and management of menstrual disorders. We have added this limitation and identified clinical recognition of abnormal/heavy menstrual bleeding, anemia, possible underlying conditions, counseling, management, and referral as priorities for future educational interventions. We also revised the manuscript throughout to replace strong causal/evaluative terms such as 'transformed,' 'validated,' and uniform claims of improvement with more appropriate wording. | L826–882; L884–900 |
| **Abstract**   - Suggest mentioning midwifery students, in addition to nursing, at least once in your title or abstract so that this inclusion criterion is clear.   Methods:   - Suggest reporting the post-evaluation timepoint as '6 months post-intervention' rather than calendar dates, as the current phrasing is difficult to follow (e.g. the intervention occurred over 4 weeks, yet the total study duration was 6 months). - Suggest defining the WASH abbreviation for clarity, even though it may be a familiar term to most readers in this field.   Results:   - The reported mean values do not have much meaning on their own as there are no descriptors of the scores or a possible range reported. Suggest adding some context or refining so results are more meaningful. - On line 48, please amend to state “average menstrual cycle length” - The phrase 'attitudes toward menstruation in anticipation and prediction of menstruation' is unclear, as it's not evident what construct is being measured (e.g., attitudes about predictability of one's cycle? anticipatory anxiety?). Suggest refining this wording for clarity   Conclusion:   - “*hereafter referred to as the PE intervention*” is not needed, as you do not use the PE abbreviation again in the abstract. You also previously defined this term in the methods section. - In addition to the single-site limitation already noted, the study also had only one post-test evaluation, so the sustainability of any effects remains unclear (even though the evaluation occurred at 6 months, as this could be significantly lower than if testing was done immediately post). This should be amended or acknowledged in the abstract. | Thank you. We revised the Abstract throughout. Midwifery students are now explicitly identified; the follow-up is described as 6 months post-intervention; WASH is defined as water, sanitation, and hygiene; the MAQ score range (1-7) is provided for the reported attitude means; 'average menstrual cycle length' is used; and the unclear attitude wording was revised to describe preparedness for and predictability of menstruation. The Results now clearly describe the reported values as endline between-group comparisons. We also shortened the Conclusion, removed unnecessary PE wording, and acknowledged that a single post-intervention assessment cannot establish the sustainability or trajectory of the observed changes. | L28–67 |
| **INTRODUCTION:**  **general comments**  Overall, the introduction is lengthy given the amount of background needed to orient the reader, and some details may fit better elsewhere: for example, study setting information (starting line 137) could move to the methods section, and intervention design details (page 7) could also move to methods or be removed altogether to avoid repetition. Similarly, content such as limitations related to the single-site design may be better suited to the discussion, and additional background detail could be moved to a supplement if not essential to the core narrative. Suggest refining and aiming for a more concise introduction, as public health manuscripts often favor this. | Thank you. We shortened the Introduction and removed or relocated material that was not essential to establishing the study rationale. Study-setting information was moved to the Methods, and the detailed intervention/theoretical material was consolidated in the Methods/Theoretical Framework section to reduce repetition and focus the Introduction more directly on the evidence gap and study objective. | L81–189; L219–247 |
| **Lines 111 -112**   1. Although the meaning becomes clearer later in the manuscript, the term ‘hostel settings’ may not be familiar to all readers at first use. Suggest including a definition or refining the sentence for clarity. 2. Do you have references demonstrating this purported cultural silence broadly across academic settings? If not, suggest narrowing the claim's scope, for example, specifying that it refers to academic and hostel settings in Bangladesh, and/or to the nursing field specifically, rather than academic settings in general. | Thank you. At first use, we now clarify that student hostels are residential accommodation for students. We also narrowed the statement about menstrual silence to the relevant Bangladeshi nursing/academic and residential context rather than presenting it as a universal feature of academic settings. | L116–121 |
| **Lines 111-113**  Suggest adding ‘and their own menstrual health’, as you note on line 120 | Thank you. We revised the sentence to acknowledge both students' future professional responsibilities and their own menstrual health. | L116–121 |
| **Line 123**  The referent of "both groups" is unclear, please clarify which two groups this refers to (e.g., nursing and midwifery students, intervention and control groups, or another pairing). | Thank you. We replaced the ambiguous phrase 'both groups' with 'nursing and midwifery students' so that the referent is explicit. | L131–135 |
| **Lines 124-125**  The claim that nursing and midwifery students are a "crucial but under-examined population in menstrual health research” is a strong empirical statement that warrants a supporting citation (e.g., a systematic review or bibliometric analysis demonstrating this gap). As written, it's unclear what evidence supports this characterization. Suggest either citing a source that substantiates this gap, or narrowing the claim to a more specific and verifiable scope, for example, "under-examined in the context of a  specific outcome/intervention type/geographic setting." | Thank you. We narrowed this claim to the specific evidence gap addressed by the study, stating that evidence on educational interventions addressing MHH among nursing and midwifery students in Bangladesh remains limited, rather than describing this population broadly as 'under-examined.' Relevant supporting citations were also added where appropriate. | L133–145 |
| **Lines 130-133**  Suggest including a reference to support the claimed "multiplier effect" of MHH literacy | Thank you. We revised the statement and added supporting literature for the potential broader educational role of menstrual health literacy, while avoiding an unsupported claim of a definite 'multiplier effect.' | L141–147 |
| **Lines 165-167**  Please provide a reference to support the claim that group dialogue and role-play activities reduce embarrassment. This may not hold universally as for some students, these activities could increase discomfort, particularly given the stigmatized nature of the topic. Consider citing evidence or acknowledging this as a possible limitation/individual variation. | Thank you. We revised this paragraph, Rather than stating that group dialogue and role-play necessarily reduce embarrassment, we now state that these activities were used to provide opportunities for discussion, reflection, and experience-sharing. We also acknowledge that individual responses may vary and that some students may experience discomfort when discussing a sensitive and stigmatized topic. | L–239-243 |
| **Study Design**  The manuscript describes the overall design as quasi-experimental (i.e., non-randomized), which appears to refer to how students were assigned to intervention versus control groups. However, the text also states that randomization occurred by study year, with Figure 2 reporting stratified random sampling. This is a potentially important distinction that isn't currently clear: stratified random sampling of participants within each study-year stratum (which affects who was measured) is different from random assignment of groups to intervention or control (which affects internal validity/causal inference). As currently written and depicted in Figure 2, it reads as though randomization may have addressed the quasi-experimental limitation, when it may only pertain to participant selection rather than group assignment. Please clarify explicitly: (1) how students/class sections were assigned to intervention vs. control (and whether this was random or not), and (2) how the stratified random sampling by study year fits into that process. This distinction has direct implications for how causal claims in the results/discussion should be framed. | Thank you for highlighting this important distinction. We now explicitly separate participant selection from intervention allocation. Students were stratified by academic year and randomly selected within strata from official attendance registers; this sampling procedure determined who was included in the study and did not determine intervention exposure. Each academic-year class had two existing sections; one section was allocated to the intervention condition and the other to the control condition. Section-level allocation was non-randomized, and the study is therefore described consistently as quasi-experimental. We also acknowledge that residual measured or unmeasured differences between sections cannot be excluded. | L193–218; L269–288 |
| **Line 186**  Strongly suggest refining the statement that "meaningful causal inference" can be drawn from this pre/post design. While the addition of a control group strengthens the design relative to a single-group pre/post study, this was not an RCT, nor a controlled interrupted time series with multiple evaluations, and the study was conducted at a single hostel institution, which raises contamination and confounding risks discussed elsewhere in this review. In addition, knowledge, attitudes, and practices are self-reported and may be subject to social desirability bias, particularly among students who may feel pressure to report what they perceive is expected of them post-intervention. | Thank you. We removed the statement that the design permitted 'meaningful causal inference' and moderated causal language throughout the manuscript. The revised Methods and Discussion explicitly acknowledge the non-randomized, single-site design, possible contamination and residual confounding, and the potential for recall and social desirability bias arising from self-reported KAP outcomes. | L193–218; L802–868 |
| **Lines 213-215 and 232-235**  These statements would be more appropriate under limitations in the discussion | Thank you. These statements were removed from the Methods and the relevant considerations were incorporated into the Discussion/Limitations section. | L802–868 |
| **Lines 216-217**  The reported ages of nursing students are a study result (i.e., a  characteristic of the enrolled sample) and would typically be reported in the results section, not methods, unless a specific age range was a pre-specified eligibility/inclusion criterion, in which case that criterion (not the sample's actual observed ages) belongs in methods. | Thank you. The observed participant ages were removed from the Methods and are reported with the participant characteristics in the Results. | L473–484; Table 2 |
| **Line 240**  The response rate of 96.7% is a study result and would be more appropriate in the results section rather than methods. | Thank you. The 96.7% response rate was moved from the Methods to the Results section, where participant recruitment and flow are reported. | L471–472 |
| **Section 2.3**  Please include a reference for G*Power as you have done for JMP Pro on line 352 | Thank you. We added the standard G*Power citation in the Sample Size Determination section. | L314–328 |
| **Line 337**  It is unclear why interviewer bias is listed as a concern, given that self-administered surveys were used. Interviewer bias typically applies to interviewer-administered data collection. Please clarify whether this refers to a different aspect of data collection (e.g., bias introduced during survey distribution or clarification of questions) or remove/revise if not applicable. | Thank you. We removed the inappropriate reference to interviewer bias. The revised text clarifies that the questionnaires were self-administered and that trained supervisors provided standardized procedural instructions without influencing participants' responses. | L401–404 |
| **Lines 349-350**  Please clarify why daily questionnaire checks for completeness were conducted, given that the study design included only single pre- and post-test assessments. If data collection occurred over multiple days within each timepoint (e.g., surveys administered across a data collection window rather than a single day), please specify this in the text. | Thank you. We clarified that data collection at each assessment was conducted across scheduled class sessions/data-collection days, and that completed questionnaires were reviewed for completeness at the end of each data-collection day before secure storage and data entry. | L418–420 |
| **Line 353**  Please provide more explanation/rationale for removing outliers, specifically, what statistical criteria or threshold defined an "outlier" (e.g.,  ±3 SD, IQR method), how many data points were removed, and whether this decision was pre-specified or made post hoc after seeing the data. Post hoc outlier removal without a clear, pre-specified rule can introduce bias into the results. | Thank you. We removed the statement indicating that statistical outliers were removed. The revised data-management description focuses on completeness, coding, logical/range checks, and verification of implausible values rather than post hoc exclusion based solely on statistical extremity. | L421–426 |
| **Section 3.1**  Suggest reducing the text here to focus on the most important participant characteristics and then refer the reader to Table 1 rather than describing all demographics in prose. | Thank you. Section 3.1 was shortened to report only the most important participant characteristics, with readers referred to Table 2 for the full socio-demographic profile. | L473–484 |
| **Table 1**  Approximately one-quarter of the sample did not reside in a hostel, yet the methods describe the intervention as adapted specifically for students in a hostel environment.   1. Were any sub-analyses conducted comparing hostel vs. non-hostel residents, or is this discrepancy addressed elsewhere? If not, please consider discussing whether/how non-hostel residents' inclusion affects the intervention's relevance or the interpretation of results for this subgroup. 2. Some variables do not sum to the reported total (n=192). Based on the questionnaires in the supplement, this discrepancy may reflect unreported categories (e.g., "other" religion) and/or missing data. Please confirm the reported n for each variable and/or provide additional detail in the supplement (e.g., missing data counts per variable) for clarity and transparency. | Thank you. We clarified that the intervention content was relevant to both hostel and non-hostel students, although some components related to shared WASH facilities and residential environments may have differed in relevance. No hostel-versus-non-hostel subgroup analysis was conducted, and this is now acknowledged as a limitation and future research direction. We also rechecked Table and revised categories/counts so that the reported distributions are transparent and sum appropriately to the group totals, including the previously omitted 'other' category where applicable. | Table 2; L837–842 |
| **Table 2**   1. Strongly suggest relabeling the variable "*correct age at menarche (13 years)*." Menarche onset varies normally across individuals and populations, and typical onset has also changed over time (Papadimitriou, 2016, J Pediatr Adolesc Gynecol, DOI: 10.1016/j.jpag.2015.12.002). There is nothing inherently "correct" about 13 years specifically and framing it this way risks reinforcing the misconception among nurses, girls, and women that menarche outside this age is abnormal or "incorrect." Consider relabeling as something like "knowledge of typical/average age range at menarche" and clarifying in the text what age or range was used as the reference answer and why. 2. Please strongly consider including a column showing the within-group McNemar p-value for the control group as well. This is particularly important given that, for a couple of variables, the control group showed a greater absolute change pre/post than the intervention group. Reporting the McNemar result only for the intervention group is a form of selective or outcome reporting bias, and it obscures the observed changes in the control group. As currently presented, this could be misinterpreted to imply that control-group changes were not significant, when the between-group p-values show that only 2 variables remained significant overall. Please either include the within-group McNemar column for both groups, or remove it for the intervention group alone. | Thank you. We relabeled the menarche item to avoid implying that 13 years is the only 'correct' age and clarified that it was the prespecified reference response for the average age at menarche, not a threshold of normality. We also revised the presentation of within-group analyses to avoid selective reporting. | Table 3; L510–542 |
| **Table 3**   1. Consider adding a footnote defining the potential range of scores (1-7) to improve clarity 2. Consider reporting median and IQR in addition to means, given that these are ordinal (1-7 scale) variables — median/IQR may better reflect central tendency and spread if distributions are skewed or show ceiling/floor effects. This could be included in the main table or supplement. 3. Suggest relabeling or adding footnotes to clarify the "anticipation and prediction" and "denial of effect" variable labels as their meaning is not immediately clear from the label alone. | Thank you. We added the potential MAQ score range (1-7) and explanatory footnotes for the 'anticipation/prediction' and 'denial of effect' subscales. Mean +/- SD was retained because the prespecified inferential analyses were based on subscale scores using t-tests; We added median and IQR. we clarified the scale and interpretation and avoided implying that higher scores uniformly represent more positive attitudes across all conceptually distinct MAQ domains. | Table 4; L543–575 |
| **Lines 473-479**  Increased product access, rather than the educational content itself, could plausibly explain some of the observed increases, since the intervention group was given sample menstrual products, and it's unclear whether these were retained after the demonstration. Additionally, if control group participants used different product types (e.g., menstrual cups) at different rates, the "changing materials ≥3 times/day" indicator may not be an equally valid practice marker across product types. Please clarify sample product retention and whether this indicator is appropriately interpreted across products. As product-type data appear to have been collected for each participant (based on supplement), consider adjusting for product type as a covariate in the analysis to account for this potential confound. | Thank you. We revised the Results and Discussion to acknowledge that provision/exposure to sample menstrual products may have contributed to the observed increase in disposable-pad use and that this change should not be attributed solely to educational content. We also clarified that changing menstrual materials >=3 times/day is a descriptive management indicator rather than a universally appropriate hygiene threshold because appropriate frequency depends on menstrual flow and product type. Product type was not included as a covariate in the primary analysis; therefore, residual confounding related to product choice is now explicitly acknowledged as a limitation. | L577–601; L729–745; L842–848 |
| **Lines 499-503**  This statement is too strong, as some domains showed non-significant between-group changes, particularly among knowledge indicators.  Suggest refining (e.g., "the PE intervention was associated with significant improvements across multiple variables within the knowledge, attitudes, and practice domains" rather than implying uniform change across all domains). | Thank you. We revised the statement to indicate that the PE intervention was associated with significant changes across multiple variables within the knowledge, attitude, and practice domains, while explicitly acknowledging that the magnitude and statistical significance varied across individual indicators. | L641–648 |
| **Lines 503-508**  These statements seem a bit strong for a single institution pre/post study, please refine for accuracy | Thank you. We moderated these statements throughout the Discussion to reflect the single-institution, non-randomized quasi-experimental design and now use associative rather than causal language. | L648–658; L884–900 |
| **Lines 535**  Again, this statement seems too strong for what the data in Table 3 support. Of the 5 variables assessed, only 3 showed significant between-group differences, and the effect size for these was medium, not large or "profound." Language such as "associated with" would be a more accurate descriptor than "transformed," particularly given the single-center, pre/post design. | Thank you. We revised Section 4.2 to describe the intervention as being 'associated with' changes in selected attitude dimensions, removed terms such as 'transformed' and 'profound,' and aligned the interpretation with the observed pattern of three statistically significant between-group MAQ domains rather than implying uniform attitudinal change. | L685–716 |
| **Section 4.2**  It would also be informative to discuss possible reasons why 2 of the 5 variables did not reach statistical significance, rather than focusing discussion solely on the significant findings. | Thank you. We expanded Section 4.2 to discuss the two MAQ domains without statistically significant between-group differences. The revised discussion considers the multidimensional nature of menstrual attitudes, menstrual symptoms and personal experiences, social/environmental influences, participants' prior health education, and the possibility that some attitudes require longer or more intensive intervention to change. | L685–716 |
| **Section 4.3**  As noted above, please clarify whether these changes could be at least partially attributed to the intervention group's access to distributed supplies, which may have made certain practices more directly achievable regardless of any change in knowledge or attitudes.  Relatedly, based on the survey questions in the supplement, did product use differ within each group between pre- and post-test? This is worth considering even independent of physical product distribution: if the intervention content characterized disposable pads as a superior product, this messaging alone could have shifted participants' product choices or their self-reported responses, separate from any change in underlying knowledge or attitudes. Ideally, the analysis should adjust for product type as a covariate to help disentangle these effects from the intervention's informational content.  Lastly, the heading and text (lines 556-557) state that the observed changes demonstrate improved self-efficacy. However, self-efficacy is a distinct, well-defined construct, and while improvements in KAP may suggest enhanced capability or readiness to engage in the target behavior, they do not independently establish increased self-efficacy, which does not appear to have been directly measured in this study. The following references provide more information: https://pubmed.ncbi.nlm.nih.gov/27280365/ https://pubmed.ncbi.nlm.nih.gov/10938914/ | Thank you. Section 4.3 was substantially revised. We now acknowledge the potential influence of distributed/sample products and product exposure on reported practices; clarify that changing frequency must be interpreted in relation to menstrual flow and product type; and acknowledge that product type was not included as a covariate in the primary analysis. We also changed the section heading to 'Changes in Menstrual Management Practices' and removed claims of improved self-efficacy because self-efficacy was not directly measured. The revised interpretation is limited to observed self-reported practices and possible capability/readiness to adopt selected behaviors. | L717–745; L842–848 |
| **Lines 561-562**  This statement indicates significant between-group improvement in sun-drying practices; however, this does not appear to be supported by the between-group results in Table 3. Please review and correct this discrepancy. | Thank you. We checked this statement against the reported table values and revised the text so that the description of sun-drying practices is consistent with the reported between-group results and does not overstate the finding. | L596–598 |
| **Lines 564-567**  Please address whether other factors, such as the provision of materials, may have also contributed to these outcomes. Also, since there was only a single post-test measurement (albeit at a meaningful 6-month follow-up) it may be overly strong to state that the behavioral pathway for sustainable hygiene behaviors has been "validated." Consider softer language (e.g., "these findings provide preliminary support for..." or "suggest a potential pathway toward...") that better reflects the single-timepoint design. | Thank you. We now explicitly acknowledge that product provision, product exposure/choice, and environmental conditions may have contributed to the observed practice differences. We also removed the statement that the behavioral pathway was 'validated' and replaced it with cautious wording indicating that the findings are consistent with, or provide preliminary support for, the proposed pathway. We further acknowledge that one six-month post-test cannot establish sustainability. | L729–745 |
| **Limitations**  Suggest adding the following limitations:   1. This study did not include any male nursing students, who may have different (or less) baseline knowledge and attitudes related to menstrual hygiene management (MHH), yet may still provide MHH-related care or education to patients in future clinical practice. This is worth noting as a limitation affecting the generalizability of findings to the full nursing/midwifery student population. 2. As previously noted, the limitations section should also discuss the educational intervention's scope, specifically, its lack of coverage of clinically oriented content such as recognition of atypical menstrual health (e.g., prolonged bleeding, heavy menstrual bleeding, and associated risks such as anemia or bleeding disorders). | Thank you. Both limitations have been added. We now note that male nursing students were not included, limiting generalizability to the full nursing/midwifery student population. We also clarify that the intervention focused mainly on personal menstrual health KAP and did not comprehensively address clinical assessment and management of menstrual disorders, including prolonged/heavy menstrual bleeding, anemia, bleeding disorders, and other potential underlying conditions. These areas are identified as priorities for future educational interventions. | L826–882 |
| **Lines 573-582**  I could not identify any results or survey questions relating to students' confidence discussing menstruation or readiness to discuss it with future patients. Were these anecdotal reports rather than measured outcomes? If so, please clarify the source of this data. Also, suggest revising the phrases "this transformation is significant" and "building institutional educational capacity for gender-sensitive nursing education," as both interpretations appear to go beyond what was actually measured in this study. | Thank you. Confidence in discussing menstruation, readiness to counsel future patients, and institutional educational capacity were not directly measured. We therefore removed statements presenting these as study findings, including the stronger language regarding 'transformation.' The revised paragraph focuses on the measured KAP outcomes and identifies communication skills, counseling confidence, clinical competencies, and patient-care behaviors as outcomes for future research. | L760–770 |
| **Fig 1**  Suggest revising this figure as:   1. The mechanisms depicted are unclear. For example, how do "human resources" reduce stigma, and how do "participant characteristics" lead to increased hygiene behavior? Passive resources, inherent participant traits, and environment alone likely do not bring about change on their own, which seems to be precisely why a participatory educational (PE) intervention was used, but this reasoning isn't reflected in the current framework. 2. The PE intervention is not clearly evident in this figure 3. Consider using "improved hygiene KAP" rather than "sustainable hygiene behaviour," or positioning KAP as an intermediate outcome rather than an endpoint. Additionally, "supporting institutional culture" is listed as an outcome but does not appear to have been measured in this study.   One option to address these issues would be to use an implementation science causal pathway diagram (Klasnja et al., 2024, Implement Res Pract, DOI: 10.1177/26334895241248851) to explicitly map the intervention, its theorized mechanism(s) of action, anticipated proximal and distal outcomes, necessary pre-conditions, and relevant moderators. This would clarify the causal logic connecting the PE intervention to hypothesized outcomes more precisely than the current figure. | Thank you. Figure 1 was redesigned to make the intervention logic explicit. The revised framework identifies the PE intervention and its participatory components, theorized cognitive/affective/behavioral mechanisms, measured proximal KAP outcomes, relevant contextual moderators/preconditions, and potential distal clinical/professional outcomes that were not measured. Unmeasured 'supporting institutional culture' was removed as a study outcome, and the figure no longer presents sustainable behavior as an established endpoint. | Fig. 1; L220–247 |
| **Fig 2**  Please see comments above under study design | Thank you. Figure 2 was revised to clearly distinguish stratified random participant selection from non-randomized class-section allocation and to show participant flow through eligibility/approach, enrollment, allocation, six-month follow-up, and analysis. The figure now reflects 397 students approached, 384 enrolled (192 per group), and 192 per group completing follow-up and analysis, with no attrition. | Fig. 2 (L312); L193–205 |
| **Supplement**  Please double check your supplemental tables as it appears some formatting may need fixing (e.g. duplicate numbering in the MAQ table) | Thank you. We carefully reviewed and corrected the Supporting Information, including duplicate numbering and formatting in the MAQ material, and updated the corresponding in-text citations/captions for consistency. | Supporting Information (S1) |
|  |  |  |
| **Reviewer #2:** |  |  |
| Reviewer #2: • Please clarify whether the reported changes represent within-group changes, between-group differences, or both, particularly in the Abstract and Results. | Thank you. We revised the Abstract and Results to distinguish within-group changes from between-group comparisons. In particular, the values highlighted in the Abstract are now explicitly described as endline intervention-versus-control comparisons rather than being labeled as within-group 'improvements.' |  |
| • The Introduction could be slightly shortened by removing some repetitive statements and focusing more directly on the knowledge gap addressed by this study. | Thank you. We shortened the Introduction by removing repetitive background material and relocating detailed setting/intervention content to the Methods. The revised Introduction focuses more directly on the evidence gap and study rationale. | L81–189 |
| • Please provide a clearer explanation of how classes were allocated to the intervention and control groups and discuss the possibility of differences between classes. | Thank you. We now explain that each academic year comprised two existing class sections, with one section allocated to the intervention condition and the other to the control condition. Allocation was non-randomized and occurred at the section level. We also acknowledge that pre-existing measured or unmeasured differences between sections cannot be completely excluded. | L193–205; L269–288 |
| • Since allocation was conducted at the class level, please clarify whether any clustering effect was considered in the statistical analysis. | Thank you. We clarified that allocation occurred at the class-section level but the primary analyses were conducted at the individual-student level and did not explicitly adjust standard errors for within-section clustering. Given the small number of section-level clusters, reliable estimation of cluster-level variance was limited. This is now stated in the Statistical Analysis and Limitations sections, and the precision and statistical significance of estimates are interpreted cautiously. | L424–426; L833–837 |
| • Please provide a little more information on how the questionnaire items were adapted from the original instruments and whether their validity was assessed after adaptation. | Thank you. We expanded Section 2.4 to describe how items were selected from the source instruments, adapted using locally familiar wording/terminology while retaining their intended constructs, reviewed by three experts for content relevance and cultural appropriateness, and pilot-tested among 30 nursing students from another college. We report internal consistency of the adapted instrument and clarify that a separate formal psychometric validation such as factor analysis was not undertaken after adaptation. | L329–357 |
| • A brief table describing the four intervention sessions, including objectives, main topics, activities, and duration, would improve the reproducibility of the intervention. | Thank you. We added a table summarizing all four intervention sessions, including each session's objective, main topics, participatory activities, and duration (60-90 minutes), to improve transparency and reproducibility. | Table 1; L358–390 |
| • The six-month interval between the intervention and endline assessment is important. Please explain more clearly why this follow-up period was selected and how it relates to assessing retention of knowledge and behavior. | Thank you. We added the rationale for the six-month follow-up. The interval was selected to assess whether differences were observable beyond the immediate post-intervention period and after participants had opportunities to apply the content during subsequent menstrual cycles. We also clarify that, because there were no immediate or intermediate post-tests, the trajectory and longer-term sustainability of change cannot be determined. | L393–400 |
| • Because multiple knowledge, attitude, and practice outcomes were tested, please clarify whether these outcomes were predefined and whether any consideration was given to the increased risk of type I error. | Thank you. We clarified that the KAP outcomes were predefined on the basis of the questionnaire and intervention content. No formal multiple-testing adjustment was applied; therefore, the possibility of type I error is acknowledged. P-values are now interpreted alongside effect estimates and 95% confidence intervals. | L449–452 |
| • There was a statistically significant baseline difference in age between the two groups (p=0.048). Please discuss whether this difference could have influenced the observed outcomes and whether adjustment for age was considered. | Thank you. We now report and discuss the small baseline age difference (22.5 +/- 3.2 vs. 23.1 +/- 2.7 years; mean difference -0.6 years, 95% CI -1.19 to -0.01; SMD -0.20; p=0.048). Because the magnitude was small, age was not included as a covariate in the primary unadjusted analyses; however, we explicitly acknowledge that residual confounding by age cannot be excluded. | Table 2; L473–500; L826–868 |
| • Please report effect sizes and confidence intervals consistently for the major intervention outcomes where possible, rather than relying mainly on p-values. | Thank you. We revised the Results tables and Statistical Analysis section to report effect estimates and 95% confidence intervals more consistently. Categorical knowledge/practice outcomes are accompanied by absolute between-group differences in proportions and 95% CIs, while MAQ outcomes include mean differences with 95% CIs and standardized effect sizes (Cohen's d) where appropriate. | L427–452; Tables 2–5 |
| • Some statements in the Results appear to include interpretation. Consider moving explanatory or interpretive statements to the Discussion. | Thank you. We reviewed the Results and removed or relocated explanatory/interpretive statements to the Discussion. The Results now focus on the observed data, effect estimates, confidence intervals, and statistical comparisons. | L473–620 |
| • Given the quasi-experimental, non-randomized design and single-institution setting, the causal language throughout the manuscript should be slightly moderated. | Thank you. We moderated causal language throughout the Abstract, Results, Discussion, and Conclusion. Terms implying causation or uniform transformation were replaced with wording such as 'was associated with,' 'observed differences,' and 'suggests,' consistent with the non-randomized single-site design. | Throughout manuscript; L884–900 |
| • Please expand the discussion of the study's generalizability, particularly because the study was conducted in a single nursing college. | Thank you. We expanded the generalizability discussion to emphasize that the study was conducted at a single government nursing institution and may not fully represent students in other regions, institutional settings, or socio-cultural contexts in Bangladesh. We also identify multi-site studies as necessary for confirmation. | L826–868 |
| • Please check the consistency of the numerical values reported in the text against Tables 1–4. | Thank you. We rechecked the numerical values reported in the Abstract, Results text, and Tables 1-4 and corrected inconsistencies so that the narrative and tabulated results are aligned. | L48–67; L473–620; Tables 2–5 |
| • The participant flow could be presented more clearly, showing the number assessed for eligibility, enrolled, allocated to each group, completing follow-up, and included in the final analysis. | Thank you. We revised the participant-flow description and Figure 2 to show the number approached/eligible (n=397), enrolled (n=384), allocated to the intervention and control groups (n=192 each), completing the six-month follow-up (n=192 each), and included in the final analysis (n=192 each). | Fig. 2 (L312); L471–472 |
| • The Discussion could better distinguish the findings of the present study from findings reported in previous studies and explain possible reasons for similarities or differences. | Thank you. We revised the Discussion to distinguish the present study findings from previous research and added cautious explanations for similarities and differences, including contextual, educational, environmental, and product-related factors where relevant. | L659–801 |
| • The Conclusion could be shortened slightly and focus mainly on the key findings, while emphasizing that confirmation in larger and multiple-site studies is needed. | Thank you. We shortened the Conclusion to focus on the key observed findings and the limitations of the single-site quasi-experimental design. It now emphasizes the need for larger, multi-site studies with stronger designs and repeated follow-up assessments to confirm the findings, assess generalizability, and determine whether changes are sustained and translate into clinical practice. | L884–900 |
|  |  |  |
| **Reviewer #3:** |  |  |
| Reviewer #3: The manuscript is well-written and structured. The statistical analysis needs to be revised for in-depth understanding. An ANCOVA test can be added to the analysis for comparing the invention and control groups. | Thank you for this suggestion. We considered the recommendation. However, the Academic Editor explicitly indicated that the recommendation to add ANOVA/ANCOVA could be disregarded. We therefore retained the prespecified analyses and instead strengthened reporting of effect estimates, 95% confidence intervals, baseline imbalance, and design limitations. | L421–452 |
| - In line 100, “menstruation is often treated as a secretive and impure phenomenon” the wording “secretive and impure” needs to be improved | Thank you. We revised the sentence to use more neutral and culturally sensitive wording: menstruation is now described as being 'surrounded by secrecy and culturally embedded notions of impurity' rather than as a 'secretive and impure phenomenon.' | L100–104 |
| - In the method section, please clarify how 100% retention rate was achieved (line 343-345) | Thank you. We expanded the Data Collection Procedure to explain the retention process. Follow-up assessments were coordinated with scheduled academic activities, participant identification codes and attendance records were used to track participation, and students who missed intervention sessions were contacted through class representatives and offered make-up sessions. These procedures facilitated continued participation, and all 384 baseline participants completed the six-month follow-up. | L407–415 |
| - It is recommended to mention the IRB approval registration number and details. | Thank you. We added the name of the approving ethics/IRB body and the corresponding approval/registration number in the Methods ethics statement. | L453–457 |
| - Define PE (line 144) | Thank you. Participatory Education (PE) is now defined at its first relevant occurrence in the Methods as an interactive, learner-centered approach involving active engagement through discussion, reflection, role-play, practical demonstration, problem-solving, and peer learning. We also cite the WaterAid Menstrual Hygiene Matters training guide on which the participatory approach was based. | L193–205; L219–246 |
| - Relying on t-test is an outdated approach. It is recommended to perform ANCOVA test. | Thank you for the suggestion. As noted above, the Academic Editor specifically indicated that the recommendation to add ANOVA/ANCOVA could be disregarded. We therefore did not add ANCOVA and retained the prespecified statistical approach, while strengthening reporting of effect sizes, 95% confidence intervals, baseline differences, and the limitations of the non-randomized design. | L421–452 |
