# Supplementary material for: Effectiveness of a participatory educational intervention on menstrual health and hygiene knowledge, attitudes, and practices among nursing and midwifery students in Bangladesh
Source: PLoS One. 2026 Nov 10;21(11):e0362603. doi: 10.1371/journal.pone.0362603 (PMC13750551; doi:10.1371/journal.pone.0362603)
Supplement: Peer review history

# Peer review: Decision Letter 0

**DOI:** 10.1371/journal.pone.0362603.r001

**Authors:** Alison Parker

1 Oct 2026

Dear Dr. Yamauchi,

Please submit your revised manuscript by Nov 30 2026 11:59PM. If you will need more time than this to complete your revisions, please reply to this message or contact the journal office at plosone@plos.org. When you're ready to submit your revision, log on to https://www.editorialmanager.com/pone/ and select the 'Submissions Needing Revision' folder to locate your manuscript file.

- A letter that responds to each point raised by the academic editor and reviewer(s). You should upload this letter as a separate file labeled 'Response to Reviewers'.
- A marked-up copy of your manuscript that highlights changes made to the original version. You should upload this as a separate file labeled 'Revised Manuscript with Track Changes'.
- An unmarked version of your revised paper without tracked changes. You should upload this as a separate file labeled 'Manuscript'.

If you would like to make changes to your financial disclosure, please include your updated statement in your cover letter. Guidelines for resubmitting your figure files are available below the reviewer comments at the end of this letter.

If applicable, we recommend that you deposit your laboratory protocols in protocols.io to enhance the reproducibility of your results. Protocols.io assigns your protocol its own identifier (DOI) so that it can be cited independently in the future. For instructions see: https://journals.plos.org/plosone/s/submission-guidelines#loc-laboratory-protocols. Additionally, PLOS One offers an option for publishing peer-reviewed Lab Protocol articles, which describe protocols hosted on protocols.io. Read more information on sharing protocols at https://plos.org/protocols?utm_medium=editorial-email&utm_source=authorletters&utm_campaign=protocols.

As the corresponding author, your ORCID iD is verified in the submission system and will appear in the published article. PLOS supports the use of ORCID, and we encourage all coauthors to register for an ORCID iD and use it as well. Please encourage your coauthors to verify their ORCID iD within the submission system before final acceptance, as unverified ORCID iDs will not appear in the published article. *Only*  the individual author can complete the verification step; PLOS staff *cannot*  verify ORCID iDs on behalf of authors.

We look forward to receiving your revised manuscript.

Kind regards,

Alison Parker

Academic Editor

PLOS One

Journal Requirements:

When submitting your revision, we need you to address these additional requirements.

1. Please ensure that your manuscript meets PLOS ONE's style requirements, including those for file naming. The PLOS ONE style templates can be found at

https://journals.plos.org/plosone/s/file?id=wjVg/PLOSOne_formatting_sample_main_body.pdf and

https://journals.plos.org/plosone/s/file?id=ba62/PLOSOne_formatting_sample_title_authors_affiliations.pdf

2. Please include a complete copy of PLOS’ questionnaire on inclusivity in global research in your revised manuscript. Our policy for research in this area aims to improve transparency in the reporting of research performed outside of researchers’ own country or community. The policy applies to researchers who have travelled to a different country to conduct research, research with Indigenous populations or their lands, and research on cultural artefacts. The questionnaire can also be requested at the journal’s discretion for any other submissions, even if these conditions are not met.  Please find more information on the policy and a link to download a blank copy of the questionnaire here: https://journals.plos.org/plosone/s/best-practices-in-research-reporting. Please upload a completed version of your questionnaire as Supporting Information when you resubmit your manuscript.

3. Peer review at PLOS  One is not double-blinded (https://journals.plos.org/plosone/s/editorial-and-peer-review-process). For this reason, authors should include in the revised manuscript all the information removed for blind review.

4. We note that the grant information you provided in the ‘Funding Information’ and ‘Financial Disclosure’ sections do not match.

When you resubmit, please ensure that you provide the correct grant numbers for the awards you received for your study in the ‘Funding Information’ section.

5. Thank you for stating the following financial disclosure: [This research was made possible through the support of Gates Foundation (INV-075902) grants.].

Please state what role the funders took in the study.  If the funders had no role, please state: ""The funders had no role in study design, data collection and analysis, decision to publish, or preparation of the manuscript.""

If this statement is not correct you must amend it as needed.

Please include this amended Role of Funder statement in your cover letter; we will change the online submission form on your behalf.

6. We note that you have indicated that there are restrictions to data sharing for this study. For studies involving human research participant data or other sensitive data, we encourage authors to share de-identified or anonymized data. However, when data cannot be publicly shared for ethical reasons, we allow authors to make their data sets available upon request. For information on unacceptable data access restrictions, please see http://journals.plos.org/plosone/s/data-availability#loc-unacceptable-data-access-restrictions.

Before we proceed with your manuscript, please address the following prompts:

a) If there are ethical or legal restrictions on sharing a de-identified data set, please explain them in detail (e.g., data contain potentially identifying or sensitive patient information, data are owned by a third-party organization, etc.) and who has imposed them (e.g., a Research Ethics Committee or Institutional Review Board, etc.). Please also provide contact information for a data access committee, ethics committee, or other institutional body to which data requests may be sent.

b) If there are no restrictions, please upload the minimal anonymized data set necessary to replicate your study findings to a stable, public repository and provide us with the relevant URLs, DOIs, or accession numbers. Please see http://www.bmj.com/content/340/bmj.c181.long for guidelines on how to de-identify and prepare clinical data for publication. For a list of recommended repositories, please see https://journals.plos.org/plosone/s/recommended-repositories. You also have the option of uploading the data as Supporting Information files, but we would recommend depositing data directly to a data repository if possible.

Please update your Data Availability statement in the submission form accordingly.

7. Your ethics statement should only appear in the Methods section of your manuscript. If your ethics statement is written in any section besides the Methods, please move it to the Methods section and delete it from any other section. Please ensure that your ethics statement is included in your manuscript, as the ethics statement entered into the online submission form will not be published alongside your manuscript.

8. We note that Figure 2 in your submission contain copyrighted images. All PLOS content is published under the Creative Commons Attribution License (CC BY 4.0), which means that the manuscript, images, and Supporting Information files will be freely available online, and any third party is permitted to access, download, copy, distribute, and use these materials in any way, even commercially, with proper attribution. For more information, see our copyright guidelines: http://journals.plos.org/plosone/s/licenses-and-copyright.

We require you to either (1) present written permission from the copyright holder to publish these figures specifically under the CC BY 4.0 license, or (2) remove the figures from your submission:

1. You may seek permission from the original copyright holder of Figure 2 to publish the content specifically under the CC BY 4.0 license.

We recommend that you contact the original copyright holder with the Content Permission Form (http://journals.plos.org/plosone/s/file?id=7c09/content-permission-form.pdf) and the following text:

“I request permission for the open-access journal PLOS ONE to publish XXX under the Creative Commons Attribution License (CCAL) CC BY 4.0 (http://creativecommons.org/licenses/by/4.0/). Please be aware that this license allows unrestricted use and distribution, even commercially, by third parties. Please reply and provide explicit written permission to publish XXX under a CC BY license and complete the attached form.”

Please upload the completed Content Permission Form or other proof of granted permissions as an "Other" file with your submission.

In the figure caption of the copyrighted figure, please include the following text: “Reprinted from [ref] under a CC BY license, with permission from [name of publisher], original copyright [original copyright year].”

2. If you are unable to obtain permission from the original copyright holder to publish these figures under the CC BY 4.0 license or if the copyright holder’s requirements are incompatible with the CC BY 4.0 license, please either i) remove the figure or ii) supply a replacement figure that complies with the CC BY 4.0 license. Please check copyright information on all replacement figures and update the figure caption with source information. If applicable, please specify in the figure caption text when a figure is similar but not identical to the original image and is therefore for illustrative purposes only.

8. Please include captions for your Supporting Information files at the end of your manuscript, and update any in-text citations to match accordingly. Please see our Supporting Information guidelines for more information: http://journals.plos.org/plosone/s/supporting-information.

9. If the reviewer comments include a recommendation to cite specific previously published works, please review and evaluate these publications to determine whether they are relevant and should be cited. There is no requirement to cite these works unless the editor has indicated otherwise.

[Note: HTML markup is below. Please do not edit.]

Reviewers' comments:

Reviewer's Responses to Questions

**Comments to the Author**

1. Is the manuscript technically sound, and do the data support the conclusions?

Reviewer #1: Partly

Reviewer #2: Yes

Reviewer #3: Yes

**********

2. Has the statistical analysis been performed appropriately and rigorously?

Reviewer #1: Yes

Reviewer #2: Yes

Reviewer #3: No

**********

3. Have the authors made all data underlying the findings in their manuscript fully available?

The PLOS Data policy

Reviewer #1: No

Reviewer #2: Yes

Reviewer #3: Yes

**********

4. Is the manuscript presented in an intelligible fashion and written in standard English?

Reviewer #1: Yes

Reviewer #2: Yes

Reviewer #3: Yes

**********

Reviewer #1: Dear Authors,

I hope this message finds you well.

Thank you for the opportunity to review this manuscript — menstrual hygiene management (MHH) knowledge, attitudes, and practices (KAP) among nursing and midwifery students in Bangladesh is an important and timely topic, and I appreciate the effort that has gone into this work.

In its current form, the manuscript requires revision in several areas, particularly with respect to length, methodological clarity, and the strength of causal and evaluative language used to describe the findings.

I have provided detailed comments throughout (see attached), and I want to acknowledge upfront that the volume of feedback may appear considerable. This reflects the complexity of the study design rather than a lack of merit in the work itself. I have tried, wherever possible, to explain the reasoning behind each comment and to offer concrete suggestions, with the intention of helping to strengthen the manuscript's rigor, clarity, and overall contribution. I hope the comments are received in that spirit.

Reviewer #2: • Please clarify whether the reported changes represent within-group changes, between-group differences, or both, particularly in the Abstract and Results.

• The Introduction could be slightly shortened by removing some repetitive statements and focusing more directly on the knowledge gap addressed by this study.

• Please provide a clearer explanation of how classes were allocated to the intervention and control groups and discuss the possibility of differences between classes.

• Since allocation was conducted at the class level, please clarify whether any clustering effect was considered in the statistical analysis.

• Please provide a little more information on how the questionnaire items were adapted from the original instruments and whether their validity was assessed after adaptation.

• A brief table describing the four intervention sessions, including objectives, main topics, activities, and duration, would improve the reproducibility of the intervention.

• The six-month interval between the intervention and endline assessment is important. Please explain more clearly why this follow-up period was selected and how it relates to assessing retention of knowledge and behavior.

• Because multiple knowledge, attitude, and practice outcomes were tested, please clarify whether these outcomes were predefined and whether any consideration was given to the increased risk of type I error.

• There was a statistically significant baseline difference in age between the two groups (p=0.048). Please discuss whether this difference could have influenced the observed outcomes and whether adjustment for age was considered.

• Please report effect sizes and confidence intervals consistently for the major intervention outcomes where possible, rather than relying mainly on p-values.

• Some statements in the Results appear to include interpretation. Consider moving explanatory or interpretive statements to the Discussion.

• Given the quasi-experimental, non-randomized design and single-institution setting, the causal language throughout the manuscript should be slightly moderated.

• Please expand the discussion of the study's generalizability, particularly because the study was conducted in a single nursing college.

• Please check the consistency of the numerical values reported in the text against Tables 1–4.

• The participant flow could be presented more clearly, showing the number assessed for eligibility, enrolled, allocated to each group, completing follow-up, and included in the final analysis.

• The Discussion could better distinguish the findings of the present study from findings reported in previous studies and explain possible reasons for similarities or differences.

• The Conclusion could be shortened slightly and focus mainly on the key findings, while emphasizing that confirmation in larger and multiple-site studies is needed.

Reviewer #3: The manuscript is well-written and structured. The statistical analysis needs to be revised for in-depth understanding. An ANCOVA test can be added to the analysis for comparing the invention and control groups.

**********

what does this mean?). If published, this will include your full peer review and any attached files.

If you choose “no”, your identity will remain anonymous but your review may still be made public.

**Do you want your identity to be public for this peer review?**  For information about this choice, including consent withdrawal, please see our Privacy Policy

Reviewer #1: No

Reviewer #2: No

Reviewer #3: No

**********

[NOTE: If reviewer comments were submitted as an attachment file, they will be attached to this email and accessible via the submission site. Please log into your account, locate the manuscript record, and check for the action link "View Attachments". If this link does not appear, there are no attachment files.]

To ensure your figures meet our technical requirements, please review our figure guidelines: https://journals.plos.org/plosone/s/figures

You may also use PLOS’s free figure tool, NAAS, to help you prepare publication quality figures: https://journals.plos.org/plosone/s/figures#loc-tools-for-figure-preparation.

NAAS will assess whether your figures meet our technical requirements by comparing each figure against our figure specifications.

Attachment Submitted filename: Reviewer Comments_PONE-D-26-40606.pdf

Attachment Submitted filename: review29Sept.docx

# Author comment: Author response to Decision Letter 1

**DOI:** 10.1371/journal.pone.0362603.r002

12 Oct 2026

Response to the Academic Editor and Reviewers

Manuscript ID: PONE-D-26-40606

Effectiveness of a Participatory Educational Intervention on Menstrual Health and Hygiene Knowledge, Attitudes, and Practices Among Nursing Students in Bangladesh

Dear Dr. Parker,

Thank you for the opportunity to revise our manuscript and for the thoughtful guidance from you and the reviewers. We have carefully revised the manuscript in response to the comments. In particular, we have strengthened methodological transparency, clarified participant sampling and non-randomized class-section allocation, moderated causal and evaluative language, improved reporting of effect estimates and confidence intervals, expanded the limitations, revised the figures and supporting information, and shortened the Introduction and Conclusion. Our point-by-point responses are provided below.

Responses to Academic Editor and Journal Requirements

Academic Editor - overall guidance: We carefully addressed the substantial comments from Reviewers 1 and 2 and moderated the strength of the conclusions throughout the manuscript. In accordance with your instruction, we did not add the ANOVA/ANCOVA analysis suggested by Reviewer 3. Instead, we retained the prespecified analyses and strengthened reporting of effect estimates, confidence intervals, baseline differences, and design limitations.

1. PLOS ONE style requirements: We reviewed the revised manuscript for PLOS ONE formatting and file-naming requirements and corrected the presentation accordingly.

2. Inclusivity in Global Research questionnaire: Completed. The PLOS questionnaire on inclusivity in global research has been completed and will be uploaded as Supporting Information with the revised submission.

3. Information removed for blind review: We restored the relevant author and institutional information in the revised manuscript because PLOS ONE peer review is not double-blinded.

4. Funding Information and Financial Disclosure: We reviewed and reconciled the funding information and financial disclosure so that the grant information is consistent across the manuscript and submission materials.

5. Role of the funder: We added the requested statement: 'The funders had no role in study design, data collection and analysis, decision to publish, or preparation of the manuscript.' This statement will also be included in the cover letter.

6. Data Availability: We prepared a de-identified/anonymized minimal dataset and associated metadata and deposited them in Zenodo. The Data Availability Statement has been updated accordingly: 'The anonymized dataset and associated metadata underlying the findings of this study are publicly available in the Zenodo repository at https://doi.org/10.5281/zenodo.23224070.'

7. Ethics statement: The ethics statement has been retained only in the Methods section, and the approving ethics/IRB body and approval/registration details are reported there.

8. Figure copyright: We replaced/revised the figure so that it is an original figure created by the authors and does not reproduce copyrighted third-party images. The revised figure is therefore suitable for publication under the journal's CC BY 4.0 license.

Supporting Information captions: We added captions for the Supporting Information files at the end of the manuscript and checked the corresponding in-text citations for consistency.

Previously published works suggested by reviewers: We reviewed the publications suggested by the reviewers and cited those that were relevant to the revised manuscript, including literature addressing heavy menstrual bleeding and related methodological/theoretical issues.

Point-by-Point Responses to Reviewers

We again thank the Academic Editor and reviewers for their constructive comments, which have substantially improved the clarity and rigor of the manuscript.

Sincerely,

The Authors

Reviewer #1:

Comment 1 — Reviewer comment: Reviewer #1: Dear Authors, I hope this message finds you well. Thank you for the opportunity to review this manuscript — menstrual hygiene management (MHH) knowledge, attitudes, and practices (KAP) among nursing and midwifery students in Bangladesh is an important and timely topic, and I appreciate the effort that has gone into this work. In its current form, the manuscript requires revision in several areas, particularly with respect to length, methodological clarity, and the strength of causal and evaluative language used to describe the findings. I have provided detailed comments throughout (see attached), and I want to acknowledge upfront that the volume of feedback may appear considerable. This reflects the complexity of the study design rather than a lack of merit in the work itself. I have tried, wherever possible, to explain the reasoning behind each comment and to offer concrete suggestions, with the intention of helping to strengthen the manuscript's rigor, clarity, and overall contribution. I hope the comments are received in that spirit.

Author response: We sincerely thank the reviewer for the careful, constructive, and detailed assessment of our manuscript. We have revised the manuscript extensively in response to these comments, with particular attention to methodological clarity, concise reporting, interpretation of the quasi-experimental design, and moderation of causal and evaluative language.

Revised manuscript line(s): Throughout revised manuscript

Comment 2 — Reviewer comment: Overall impression Overall, while this study includes nursing and midwifery students and discusses the potential implications of the intervention on their future clinical practice, the PE intervention appears primarily focused on students' own personal menstruation knowledge and practices, rather than preparing them as clinicians to assess menstruation clinically and recognize abnormal menstrual bleeding in patients. This includes danger signs/symptoms, how to estimate blood loss from menstrual product use, associated outcomes such as anemia, and underlying pathology (e.g., fibroids, bleeding disorders) that can contribute to heavy menstrual flow. As described, the intervention does not appear to address these clinically oriented topics, which is a gap, especially as the authors suggest that this intervention could inform potential changes to nursing curricula. Nursing and midwifery students are uniquely positioned to have these conversations with patients, and heavy menstrual bleeding is a recognized public health concern in Bangladesh (Charles et al., 2025, Am J Obstet Gynecol, DOI: 10.1016/j.ajog.2025.06.018). The authors may wish to consider incorporating content on clinical recognition and evidence-based management of heavy menstrual bleeding (e.g., Cochrane review on HMB treatment options), or, if this was intentionally outside the scope of the current intervention, please consider briefly noting this as a direction for future work in the discussion/limitations section or section 4.5. Additionally, throughout the manuscript, several statements draw stronger causal or evaluative conclusions than the study design and data appear to support (e.g., use of terms like "transformation," "validated," and claims of improvement across all measured domains). Given the quasi-experimental, single-site, single-post-test design, I'd encourage the authors to adopt more tempered language throughout and ensure that all claims are clearly grounded in the reported results. Specific instances are noted in the line-by-line comments below.

Author response: Thank you for this important overall assessment. We agree that the intervention primarily addressed students' personal menstrual health knowledge, attitudes, and management practices rather than clinical assessment and management of menstrual disorders. We have added this limitation and identified clinical recognition of abnormal/heavy menstrual bleeding, anemia, possible underlying conditions, counseling, management, and referral as priorities for future educational interventions. We also revised the manuscript throughout to replace strong causal/evaluative terms such as 'transformed,' 'validated,' and uniform claims of improvement with more appropriate wording.

Revised manuscript line(s): L826–882; L884–900

Comment 3 — Reviewer comment: Abstract Suggest mentioning midwifery students, in addition to nursing, at least once in your title or abstract so that this inclusion criterion is clear. Methods: Suggest reporting the post-evaluation timepoint as '6 months post-intervention' rather than calendar dates, as the current phrasing is difficult to follow (e.g. the intervention occurred over 4 weeks, yet the total study duration was 6 months). Suggest defining the WASH abbreviation for clarity, even though it may be a familiar term to most readers in this field. Results: The reported mean values do not have much meaning on their own as there are no descriptors of the scores or a possible range reported. Suggest adding some context or refining so results are more meaningful. On line 48, please amend to state “average menstrual cycle length” The phrase 'attitudes toward menstruation in anticipation and prediction of menstruation' is unclear, as it's not evident what construct is being measured (e.g., attitudes about predictability of one's cycle? anticipatory anxiety?). Suggest refining this wording for clarity Conclusion: “hereafter referred to as the PE intervention” is not needed, as you do not use the PE abbreviation again in the abstract. You also previously defined this term in the methods section. In addition to the single-site limitation already noted, the study also had only one post-test evaluation, so the sustainability of any effects remains unclear (even though the evaluation occurred at 6 months, as this could be significantly lower than if testing was done immediately post). This should be amended or acknowledged in the abstract.

Author response: Thank you. We revised the Abstract throughout. Midwifery students are now explicitly identified; the follow-up is described as 6 months post-intervention; WASH is defined as water, sanitation, and hygiene; the MAQ score range (1-7) is provided for the reported attitude means; 'average menstrual cycle length' is used; and the unclear attitude wording was revised to describe preparedness for and predictability of menstruation. The Results now clearly describe the reported values as endline between-group comparisons. We also shortened the Conclusion, removed unnecessary PE wording, and acknowledged that a single post-intervention assessment cannot establish the sustainability or trajectory of the observed changes.

Revised manuscript line(s): L28–67

Comment 4 — Reviewer comment: INTRODUCTION: general comments Overall, the introduction is lengthy given the amount of background needed to orient the reader, and some details may fit better elsewhere: for example, study setting information (starting line 137) could move to the methods section, and intervention design details (page 7) could also move to methods or be removed altogether to avoid repetition. Similarly, content such as limitations related to the single-site design may be better suited to the discussion, and additional background detail could be moved to a supplement if not essential to the core narrative. Suggest refining and aiming for a more concise introduction, as public health manuscripts often favor this.

Author response: Thank you. We shortened the Introduction and removed or relocated material that was not essential to establishing the study rationale. Study-setting information was moved to the Methods, and the detailed intervention/theoretical material was consolidated in the Methods/Theoretical Framework section to reduce repetition and focus the Introduction more directly on the evidence gap and study objective.

Revised manuscript line(s): L81–189; L219–247

Comment 5 — Reviewer comment: Lines 111 -112 Although the meaning becomes clearer later in the manuscript, the term ‘hostel settings’ may not be familiar to all readers at first use. Suggest including a definition or refining the sentence for clarity. Do you have references demonstrating this purported cultural silence broadly across academic settings? If not, suggest narrowing the claim's scope, for example, specifying that it refers to academic and hostel settings in Bangladesh, and/or to the nursing field specifically, rather than academic settings in general.

Author response: Thank you. At first use, we now clarify that student hostels are residential accommodation for students. We also narrowed the statement about menstrual silence to the relevant Bangladeshi nursing/academic and residential context rather than presenting it as a universal feature of academic settings.

Revised manuscript line(s): L116–121

Comment 6 — Reviewer comment: Lines 111-113 Suggest adding ‘and their own menstrual health’, as you note on line 120

Author response: Thank you. We revised the sentence to acknowledge both students' future professional responsibilities and their own menstrual health.

Revised manuscript line(s): L116–121

Comment 7 — Reviewer comment: Line 123 The referent of "both groups" is unclear, please clarify which two groups this refers to (e.g., nursing and midwifery students, intervention and control groups, or another pairing).

Author response: Thank you. We replaced the ambiguous phrase 'both groups' with 'nursing and midwifery students' so that the referent is explicit.

Revised manuscript line(s): L131–135

Comment 8 — Reviewer comment: Lines 124-125 The claim that nursing and midwifery students are a "crucial but under-examined population in menstrual health research” is a strong empirical statement that warrants a supporting citation (e.g., a systematic review or bibliometric analysis demonstrating this gap). As written, it's unclear what evidence supports this characterization. Suggest either citing a source that substantiates this gap, or narrowing the claim to a more specific and verifiable scope, for example, "under-examined in the context of a specific outcome/intervention type/geographic setting."

Author response: Thank you. We narrowed this claim to the specific evidence gap addressed by the study, stating that evidence on educational interventions addressing MHH among nursing and midwifery students in Bangladesh remains limited, rather than describing this population broadly as 'under-examined.' Relevant supporting citations were also added where appropriate.

Revised manuscript line(s): L133–145

Comment 9 — Reviewer comment: Lines 130-133 Suggest including a reference to support the claimed "multiplier effect" of MHH literacy

Author response: Thank you. We revised the statement and added supporting literature for the potential broader educational role of menstrual health literacy, while avoiding an unsupported claim of a definite 'multiplier effect.'

Revised manuscript line(s): L141–147

Comment 10 — Reviewer comment: Lines 165-167 Please provide a reference to support the claim that group dialogue and role-play activities reduce embarrassment. This may not hold universally as for some students, these activities could increase discomfort, particularly given the stigmatized nature of the topic. Consider citing evidence or acknowledging this as a possible limitation/individual variation.

Author response: Thank you. We revised this paragraph, Rather than stating that group dialogue and role-play necessarily reduce embarrassment, we now state that these activities were used to provide opportunities for discussion, reflection, and experience-sharing. We also acknowledge that individual responses may vary and that some students may experience discomfort when discussing a sensitive and stigmatized topic.

Revised manuscript line(s): L–239-243

Comment 11 — Reviewer comment: Study Design The manuscript describes the overall design as quasi-experimental (i.e., non-randomized), which appears to refer to how students were assigned to intervention versus control groups. However, the text also states that randomization occurred by study year, with Figure 2 reporting stratified random sampling. This is a potentially important distinction that isn't currently clear: stratified random sampling of participants within each study-year stratum (which affects who was measured) is different from random assignment of groups to intervention or control (which affects internal validity/causal inference). As currently written and depicted in Figure 2, it reads as though randomization may have addressed the quasi-experimental limitation, when it may only pertain to participant selection rather than group assignment. Please clarify explicitly: (1) how students/class sections were assigned to intervention vs. control (and whether this was random or not), and (2) how the stratified random sampling by study year fits into that process. This distinction has direct implications for how causal claims in the results/discussion should be framed.

Author response: Thank you for highlighting this important distinction. We now explicitly separate participant selection from intervention allocation. Students were stratified by academic year and randomly selected within strata from official attendance registers; this sampling procedure determined who was included in the study and did not determine intervention exposure. Each academic-year class had two existing sections; one section was allocated to the intervention condition and the other to the control condition. Section-level allocation was non-randomized, and the study is therefore described consistently as quasi-experimental. We also acknowledge that residual measured or unmeasured differences between sections cannot be excluded.

Revised manuscript line(s): L193–218; L269–288

Comment 12 — Reviewer comment: Line 186 Strongly suggest refining the statement that "meaningful causal inference" can be drawn from this pre/post design. While the addition of a control group strengthens the design relative to a single-group pre/post study, this was not an RCT, nor a controlled interrupted time series with multiple evaluations, and the study was conducted at a single hostel institution, which raises contamination and confounding risks discussed elsewhere in this review. In addition, knowledge, attitudes, and practices are self-reported and may be subject to social desirability bias, particularly among students who may feel pressure to report what they perceive is expected of them post-intervention.

Author response: Thank you. We removed the statement that the design permitted 'meaningful causal inference' and moderated causal language throughout the manuscript. The revised Methods and Discussion explicitly acknowledge the non-randomized, single-site design, possible contamination and residual confounding, and the potential for recall and social desirability bias arising from self-reported KAP outcomes.

Revised manuscript line(s): L193–218; L802–868

Comment 13 — Reviewer comment: Lines 213-215 and 232-235 These statements would be more appropriate under limitations in the discussion

Author response: Thank you. These statements were removed from the Methods and the relevant considerations were incorporated into the Discussion/Limitations section.

Revised manuscript line(s): L802–868

Comment 14 — Reviewer comment: Lines 216-217 The reported ages of nursing students are a study result (i.e., a characteristic of the enrolled sample) and would typically be reported in the results section, not methods, unless a specific age range was a pre-specified eligibility/inclusion criterion, in which case that criterion (not the sample's actual observed ages) belongs in methods.

Author response: Thank you. The observed participant ages were removed from the Methods and are reported with the participant characteristics in the Results.

Revised manuscript line(s): L473–484; Table 2

Comment 15 — Reviewer comment: Line 240 The response rate of 96.7% is a study result and would be more appropriate in the results section rather than methods.

Author response: Thank you. The 96.7% response rate was moved from the Methods to the Results section, where participant recruitment and flow are reported.

Revised manuscript line(s): L471–472

Comment 16 — Reviewer comment: Section 2.3 Please include a reference for G*Power as you have done for JMP Pro on line 352

Author response: Thank you. We added the standard G*Power citation in the Sample Size Determination section.

Revised manuscript line(s): L314–328

Comment 17 — Reviewer comment: Line 337 It is unclear why interviewer bias is listed as a concern, given that self-administered surveys were used. Interviewer bias typically applies to interviewer-administered data collection. Please clarify whether this refers to a different aspect of data collection (e.g., bias introduced during survey distribution or clarification of questions) or remove/revise if not applicable.

Author response: Thank you. We removed the inappropriate reference to interviewer bias. The revised text clarifies that the questionnaires were self-administered and that trained supervisors provided standardized procedural instructions without influencing participants' responses.

Revised manuscript line(s): L401–404

Comment 18 — Reviewer comment: Lines 349-350 Please clarify why daily questionnaire checks for completeness were conducted, given that the study design included only single pre- and post-test assessments. If data collection occurred over multiple days within each timepoint (e.g., surveys administered across a data collection window rather than a single day), please specify this in the text.

Author response: Thank you. We clarified that data collection at each assessment was conducted across scheduled class sessions/data-collection days, and that completed questionnaires were reviewed for completeness at the end of each data-collection day before secure storage and data entry.

Revised manuscript line(s): L418–420

Comment 19 — Reviewer comment: Line 353 Please provide more explanation/rationale for removing outliers, specifically, what statistical criteria or threshold defined an "outlier" (e.g., ±3 SD, IQR method), how many data points were removed, and whether this decision was pre-specified or made post hoc after seeing the data. Post hoc outlier removal without a clear, pre-specified rule can introduce bias into the results.

Author response: Thank you. We removed the statement indicating that statistical outliers were removed. The revised data-management description focuses on completeness, coding, logical/range checks, and verification of implausible values rather than post hoc exclusion based solely on statistical extremity.

Revised manuscript line(s): L421–426

Comment 20 — Reviewer comment: Section 3.1 Suggest reducing the text here to focus on the most important participant characteristics and then refer the reader to Table 1 rather than describing all demographics in prose.

Author response: Thank you. Section 3.1 was shortened to report only the most important participant characteristics, with readers referred to Table 2 for the full socio-demographic profile.

Revised manuscript line(s): L473–484

Comment 21 — Reviewer comment: Table 1 Approximately one-quarter of the sample did not reside in a hostel, yet the methods describe the intervention as adapted specifically for students in a hostel environment. Were any sub-analyses conducted comparing hostel vs. non-hostel residents, or is this discrepancy addressed elsewhere? If not, please consider discussing whether/how non-hostel residents' inclusion affects the intervention's relevance or the interpretation of results for this subgroup. Some variables do not sum to the reported total (n=192). Based on the questionnaires in the supplement, this discrepancy may reflect unreported categories (e.g., "other" religion) and/or missing data. Please confirm the reported n for each variable and/or provide additional detail in the supplement (e.g., missing data counts per variable) for clarity and transparency.

Author response: Thank you. We clarified that the intervention content was relevant to both hostel and non-hostel students, although some components related to shared WASH facilities and residential environments may have differed in relevance. No hostel-versus-non-hostel subgroup analysis was conducted, and this is now acknowledged as a limitation and future research direction. We also rechecked Table and revised categories/counts so that the reported distributions are transparent and sum appropriately to the group totals, including the previously omitted 'other' category where applicable.

Revised manuscript line(s): Table 2; L837–842

Comment 22 — Reviewer comment: Table 2 Strongly suggest relabeling the variable "correct age at menarche (13 years)." Menarche onset varies normally across individuals and populations, and typical onset has also changed over time (Papadimitriou, 2016, J Pediatr Adolesc Gynecol, DOI: 10.1016/j.jpag.2015.12.002). There is nothing inherently "correct" about 13 years specifically and framing it this way risks reinforcing the misconception among nurses, girls, and women that menarche outside this age is abnormal or "incorrect." Consider relabeling as something like "knowledge of typical/average age range at menarche" and clarifying in the text what age or range was used as the reference answer and why. Please strongly consider including a column showing the within-group McNemar p-value for the control group as well. This is particularly important given that, for a couple of variables, the control group showed a greater absolute change pre/post than the intervention group. Reporting the McNemar result only for the intervention group is a form of selective or outcome reporting bias, and it obscures the observed changes in the control group. As currently presented, this could be misinterpreted to imply that control-group changes were not significant, when the between-group p-values show that only 2 variables remained significant overall. Please either include the within-group McNemar column for both groups, or remove it for the intervention group alone.

Author response: Thank you. We relabeled the menarche item to avoid implying that 13 years is the only 'correct' age and clarified that it was the prespecified reference response for the average age at menarche, not a threshold of normality. We also revised the presentation of within-group analyses to avoid selective reporting.

Revised manuscript line(s): Table 3; L510–542

Comment 23 — Reviewer comment: Table 3 Consider adding a footnote defining the potential range of scores (1-7) to improve clarity Consider reporting median and IQR in addition to means, given that these are ordinal (1-7 scale) variables — median/IQR may better reflect central tendency and spread if distributions are skewed or show ceiling/floor effects. This could be included in the main table or supplement. Suggest relabeling or adding footnotes to clarify the "anticipation and prediction" and "denial of effect" variable labels as their meaning is not immediately clear from the label alone.

Author response: Thank you. We added the potential MAQ score range (1-7) and explanatory footnotes for the 'anticipation/prediction' and 'denial of effect' subscales. Mean +/- SD was retained because the prespecified inferential analyses were based on subscale scores using t-tests; We added median and IQR. we clarified the scale and interpretation and avoided implying that higher scores uniformly represent more positive attitudes across all conceptually distinct MAQ domains.

Revised manuscript line(s): Table 4; L543–575

Comment 24 — Reviewer comment: Lines 473-479 Increased product access, rather than the educational content itself, could plausibly explain some of the observed increases, since the intervention group was given sample menstrual products, and it's unclear whether these were retained after the demonstration. Additionally, if control group participants used different product types (e.g., menstrual cups) at different rates, the "changing materials ≥3 times/day" indicator may not be an equally valid practice marker across product types. Please clarify sample product retention and whether this indicator is appropriately interpreted across products. As product-type data appear to have been collected for each participant (based on supplement), consider adjusting for product type as a covariate in the analysis to account for this potential confound.

Author response: Thank you. We revised the Results and Discussion to acknowledge that provision/exposure to sample menstrual products may have contributed to the observed increase in disposable-pad use and that this change should not be attributed solely to educational content. We also clarified that changing menstrual materials >=3 times/day is a descriptive management indicator rather than a universally appropriate hygiene threshold because appropriate frequency depends on menstrual flow and product type. Product type was not included as a covariate in the primary analysis; therefore, residual confounding related to product choice is now explicitly acknowledged as a limitation.

Revised manuscript line(s): L577–601; L729–745; L842–848

Comment 25 — Reviewer comment: Lines 499-503 This statement is too strong, as some domains showed non-significant between-group changes, particularly among knowledge indicators. Suggest refining (e.g., "the PE intervention was associated with significant improvements across multiple variables within the knowledge, attitudes, and practice domains" rather than implying uniform change across all domains).

Author response: Thank you. We revised the statement to indicate that the PE intervention was associated with significant changes across multiple variables within the knowledge, attitude, and practice domains, while explicitly acknowledging that the magnitude and statistical significance varied across individual indicators.

Revised manuscript line(s): L641–648

Comment 26 — Reviewer comment: Lines 503-508 These statements seem a bit strong for a single institution pre/post study, please refine for accuracy

Author response: Thank you. We moderated these statements throughout the Discussion to reflect the single-institution, non-randomized quasi-experimental design and now use associative rather than causal language.

Revised manuscript line(s): L648–658; L884–900

Comment 27 — Reviewer comment: Lines 535 Again, this statement seems too strong for what the data in Table 3 support. Of the 5 variables assessed, only 3 showed significant between-group differences, and the effect size for these was medium, not large or "profound." Language such as "associated with" would be a more accurate descriptor than "transformed," particularly given the single-center, pre/post design.

Author response: Thank you. We revised Section 4.2 to describe the intervention as being 'associated with' changes in selected attitude dimensions, removed terms such as 'transformed' and 'profound,' and aligned the interpretation with the observed pattern of three statistically significant between-group MAQ domains rather than implying uniform attitudinal change.

Revised manuscript line(s): L685–716

Comment 28 — Reviewer comment: Section 4.2 It would also be informative to discuss possible reasons why 2 of the 5 variables did not reach statistical significance, rather than focusing discussion solely on the significant findings.

Author response: Thank you. We expanded Section 4.2 to discuss the two MAQ domains without statistically significant between-group differences. The revised discussion considers the multidimensional nature of menstrual attitudes, menstrual symptoms and personal experiences, social/environmental influences, participants' prior health education, and the possibility that some attitudes require longer or more intensive intervention to change.

Revised manuscript line(s): L685–716

Comment 29 — Reviewer comment: Section 4.3 As noted above, please clarify whether these changes could be at least partially attributed to the intervention group's access to distributed supplies, which may have made certain practices more directly achievable regardless of any change in knowledge or attitudes. Relatedly, based on the survey questions in the supplement, did product use differ within each group between pre- and post-test? This is worth considering even independent of physical product distribution: if the intervention content characterized disposable pads as a superior product, this messaging alone could have shifted participants' product choices or their self-reported responses, separate from any change in underlying knowledge or attitudes. Ideally, the analysis should adjust for product type as a covariate to help disentangle these effects from the intervention's informational content. Lastly, the heading and text (lines 556-557) state that the observed changes demonstrate improved self-efficacy. However, self-efficacy is a distinct, well-defined construct, and while improvements in KAP may suggest enhanced capability or readiness to engage in the target behavior, they do not independently establish increased self-efficacy, which does not appear to have been directly measured in this study. The following references provide more information: https://pubmed.ncbi.nlm.nih.gov/27280365/ https://pubmed.ncbi.nlm.nih.gov/10938914/

Author response: Thank you. Section 4.3 was substantially revised. We now acknowledge the potential influence of distributed/sample products and product exposure on reported practices; clarify that changing frequency must be interpreted in relation to menstrual flow and product type; and acknowledge that product type was not included as a covariate in the primary analysis. We also changed the section heading to 'Changes in Menstrual Management Practices' and removed claims of improved self-efficacy because self-efficacy was not directly measured. The revised interpretation is limited to observed self-reported practices and possible capability/readiness to adopt selected behaviors.

Revised manuscript line(s): L717–745; L842–848

Comment 30 — Reviewer comment: Lines 561-562 This statement indicates significant between-group improvement in sun-drying practices; however, this does not appear to be supported by the between-group results in Table 3. Please review and correct this discrepancy.

Author response: Thank you. We checked this statement against the reported table values and revised the text so that the description of sun-drying practices is consistent with the reported between-group results and does not overstate the finding.

Revised manuscript line(s): L596–598

Comment 31 — Reviewer comment: Lines 564-567 Please address whether other factors, such as the provision of materials, may have also contributed to these outcomes. Also, since there was only a single post-test measurement (albeit at a meaningful 6-month follow-up) it may be overly strong to state that the behavioral pathway for sustainable hygiene behaviors has been "validated." Consider softer language (e.g., "these findings provide preliminary support for..." or "suggest a potential pathway toward...") that better reflects the single-timepoint design.

Author response: Thank you. We now explicitly acknowledge that product provision, product exposure/choice, and environmental conditions may have contributed to the observed practice differences. We also removed the statement that the behavioral pathway was 'validated' and replaced it with cautious wording indicating that the findings are consistent with, or provide preliminary support for, the proposed pathway. We further acknowledge that one six-month post-test cannot establish sustainability.

Revised manuscript line(s): L729–745

Comment 32 — Reviewer comment: Limitations Suggest adding the following limitations: This study did not include any male nursing students, who may have different (or less) baseline knowledge and attitudes related to menstrual hygiene management (MHH), yet may still provide MHH-related care or education to patients in future clinical practice. This is worth noting as a limitation affecting the generalizability of findings to the full nursing/midwifery student population. As previously noted, the limitations section should also discuss the educational intervention's scope, specifically, its lack of coverage of clinically oriented content such as recognition of atypical menstrual health (e.g., prolonged bleeding, heavy menstrual bleeding, and associated risks such as anemia or bleeding disorders).

Author response: Thank you. Both limitations have been added. We now note that male nursing students were not included, limiting generalizability to the full nursing/midwifery student population. We also clarify that the intervention focused mainly on personal menstrual health KAP and did not comprehensively address clinical assessment and management of menstrual disorders, including prolonged/heavy menstrual bleeding, anemia, bleeding disorders, and other potential underlying conditions. These areas are identified as priorities for future educational interventions.

Revised manuscript line(s): L826–882

Comment 33 — Reviewer comment: Lines 573-582 I could not identify any results or survey questions relating to students' confidence discussing menstruation or readiness to discuss it with future patients. Were these anecdotal reports rather than measured outcomes? If so, please clarify the source of this data. Also, suggest revising the phrases "this transformation is significant" and "building institutional educational capacity for gender-sensitive nursing education," as both interpretations appear to go beyond what was actually measured in this study.

Author response: Thank you. Confidence in discussing menstruation, readiness to counsel future patients, and institutional educational capacity were not directly measured. We therefore removed statements presenting these as study findings, including the stronger language regarding 'transformation.' The revised paragraph focuses on the measured KAP outcomes and identifies communication skills, counseling confidence, clinical competencies, and patient-care behaviors as outcomes for future research.

Revised manuscript line(s): L760–770

Comment 34 — Reviewer comment: Fig 1 Suggest revising this figure as: The mechanisms depicted are unclear. For example, how do "human resources" reduce stigma, and how do "participant characteristics" lead to increased hygiene behavior? Passive resources, inherent participant traits, and environment alone likely do not bring about change on their own, which seems to be precisely why a participatory educational (PE) intervention was used, but this reasoning isn't reflected in the current framework. The PE intervention is not clearly evident in this figure Consider using "improved hygiene KAP" rather than "sustainable hygiene behaviour," or positioning KAP as an intermediate outcome rather than an endpoint. Additionally, "supporting institutional culture" is listed as an outcome but does not appear to have been measured in this study. One option to address these issues would be to use an implementation science causal pathway diagram (Klasnja et al., 2024, Implement Res Pract, DOI: 10.1177/26334895241248851) to explicitly map the intervention, its theorized mechanism(s) of action, anticipated proximal and distal outcomes, necessary pre-conditions, and relevant moderators. This would clarify the causal logic connecting the PE intervention to hypothesized outcomes more precisely than the current figure.

Author response: Thank you. Figure 1 was redesigned to make the intervention logic explicit. The revised framework identifies the PE intervention and its participatory components, theorized cognitive/affective/behavioral mechanisms, measured proximal KAP outcomes, relevant contextual moderators/preconditions, and potential distal clinical/professional outcomes that were not measured. Unmeasured 'supporting institutional culture' was removed as a study outcome, and the figure no longer presents sustainable behavior as an established endpoint.

Revised manuscript line(s): Fig. 1; L220–247

Comment 35 — Reviewer comment: Fig 2 Please see comments above under study design

Author response: Thank you. Figure 2 was revised to clearly distinguish stratified random participant selection from non-randomized class-section allocation and to show participant flow through eligibility/approach, enrollment, allocation, six-month follow-up, and analysis. The figure now reflects 397 students approached, 384 enrolled (192 per group), and 192 per group completing follow-up and analysis, with no attrition.

Revised manuscript line(s): Fig. 2 (L312); L193–205

Comment 36 — Reviewer comment: Supplement Please double check your supplemental tables as it appears some formatting may need fixing (e.g. duplicate numbering in the MAQ table)

Author response: Thank you. We carefully reviewed and corrected the Supporting Information, including duplicate numbering and formatting in the MAQ material, and updated the corresponding in-text citations/captions for consistency.

Revised manuscript line(s): Supporting Information (S1)

Reviewer #2:

Comment 37 — Reviewer comment: Reviewer #2: • Please clarify whether the reported changes represent within-group changes, between-group differences, or both, particularly in the Abstract and Results.

Author response: Thank you. We revised the Abstract and Results to distinguish within-group changes from between-group comparisons. In particular, the values highlighted in the Abstract are now explicitly described as endline intervention-versus-control comparisons rather than being labeled as within-group 'improvements.'

Comment 38 — Reviewer comment: • The Introduction could be slightly shortened by removing some repetitive statements and focusing more directly on the knowledge gap addressed by this study.

Author response: Thank you. We shortened the Introduction by removing repetitive background material and relocating detailed setting/intervention content to the Methods. The revised Introduction focuses more directly on the evidence gap and study rationale.

Revised manuscript line(s): L81–189

Comment 39 — Reviewer comment: • Please provide a clearer explanation of how classes were allocated to the intervention and control groups and discuss the possibility of differences between classes.

Author response: Thank you. We now explain that each academic year comprised two existing class sections, with one section allocated to the intervention condition and the other to the control condition. Allocation was non-randomized and occurred at the section level. We also acknowledge that pre-existing measured or unmeasured differences between sections cannot be completely excluded.

Revised manuscript line(s): L193–205; L269–288

Comment 40 — Reviewer comment: • Since allocation was conducted at the class level, please clarify whether any clustering effect was considered in the statistical analysis.

Author response: Thank you. We clarified that allocation occurred at the class-section level but the primary analyses were conducted at the individual-student level and did not explicitly adjust standard errors for within-section clustering. Given the small number of section-level clusters, reliable estimation of cluster-level variance was limited. This is now stated in the Statistical Analysis and Limitations sections, and the precision and statistical significance of estimates are interpreted cautiously.

Revised manuscript line(s): L424–426; L833–837

Comment 41 — Reviewer comment: • Please provide a little more information on how the questionnaire items were adapted from the original instruments and whether their validity was assessed after adaptation.

Author response: Thank you. We expanded Section 2.4 to describe how items were selected from the source instruments, adapted using locally familiar wording/terminology while retaining their intended constructs, reviewed by three experts for content relevance and cultural appropriateness, and pilot-tested among 30 nursing students from another college. We report internal consistency of the adapted instrument and clarify that a separate formal psychometric validation such as factor analysis was not undertaken after adaptation.

Revised manuscript line(s): L329–357

Comment 42 — Reviewer comment: • A brief table describing the four intervention sessions, including objectives, main topics, activities, and duration, would improve the reproducibility of the intervention.

Author response: Thank you. We added a table summarizing all four intervention sessions, including each session's objective, main topics, participatory activities, and duration (60-90 minutes), to improve transparency and reproducibility.

Revised manuscript line(s): Table 1; L358–390

Comment 43 — Reviewer comment: • The six-month interval between the intervention and endline assessment is important. Please explain more clearly why this follow-up period was selected and how it relates to assessing retention of knowledge and behavior.

Author response: Thank you. We added the rationale for the six-month follow-up. The interval was selected to assess whether differences were observable beyond the immediate post-intervention period and after participants had opportunities to apply the content during subsequent menstrual cycles. We also clarify that, because there were no immediate or intermediate post-tests, the trajectory and longer-term sustainability of change cannot be determined.

Revised manuscript line(s): L393–400

Comment 44 — Reviewer comment: • Because multiple knowledge, attitude, and practice outcomes were tested, please clarify whether these outcomes were predefined and whether any consideration was given to the increased risk of type I error.

Author response: Thank you. We clarified that the KAP outcomes were predefined on the basis of the questionnaire and intervention content. No formal multiple-testing adjustment was applied; therefore, the possibility of type I error is acknowledged. P-values are now interpreted alongside effect estimates and 95% confidence intervals.

Revised manuscript line(s): L449–452

Comment 45 — Reviewer comment: • There was a statistically significant baseline difference in age between the two groups (p=0.048). Please discuss whether this difference could have influenced the observed outcomes and whether adjustment for age was considered.

Author response: Thank you. We now report and discuss the small baseline age difference (22.5 +/- 3.2 vs. 23.1 +/- 2.7 years; mean difference -0.6 years, 95% CI -1.19 to -0.01; SMD -0.20; p=0.048). Because the magnitude was small, age was not included as a covariate in the primary unadjusted analyses; however, we explicitly acknowledge that residual confounding by age cannot be excluded.

Revised manuscript line(s): Table 2; L473–500; L826–868

Comment 46 — Reviewer comment: • Please report effect sizes and confidence intervals consistently for the major intervention outcomes where possible, rather than relying mainly on p-values.

Author response: Thank you. We revised the Results tables and Statistical Analysis section to report effect estimates and 95% confidence intervals more consistently. Categorical knowledge/practice outcomes are accompanied by absolute between-group differences in proportions and 95% CIs, while MAQ outcomes include mean differences with 95% CIs and standardized effect sizes (Cohen's d) where appropriate.

Revised manuscript line(s): L427–452; Tables 2–5

Comment 47 — Reviewer comment: • Some statements in the Results appear to include interpretation. Consider moving explanatory or interpretive statements to the Discussion.

Author response: Thank you. We reviewed the Results and removed or relocated explanatory/interpretive statements to the Discussion. The Results now focus on the observed data, effect estimates, confidence intervals, and statistical comparisons.

Revised manuscript line(s): L473–620

Comment 48 — Reviewer comment: • Given the quasi-experimental, non-randomized design and single-institution setting, the causal language throughout the manuscript should be slightly moderated.

Author response: Thank you. We moderated causal language throughout the Abstract, Results, Discussion, and Conclusion. Terms implying causation or uniform transformation were replaced with wording such as 'was associated with,' 'observed differences,' and 'suggests,' consistent with the non-randomized single-site design.

Revised manuscript line(s): Throughout manuscript; L884–900

Comment 49 — Reviewer comment: • Please expand the discussion of the study's generalizability, particularly because the study was conducted in a single nursing college.

Author response: Thank you. We expanded the generalizability discussion to emphasize that the study was conducted at a single government nursing institution and may not fully represent students in other regions, institutional settings, or socio-cultural contexts in Bangladesh. We also identify multi-site studies as necessary for confirmation.

Revised manuscript line(s): L826–868

Comment 50 — Reviewer comment: • Please check the consistency of the numerical values reported in the text against Tables 1–4.

Author response: Thank you. We rechecked the numerical values reported in the Abstract, Results text, and Tables 1-4 and corrected inconsistencies so that the narrative and tabulated results are aligned.

Revised manuscript line(s): L48–67; L473–620; Tables 2–5

Comment 51 — Reviewer comment: • The participant flow could be presented more clearly, showing the number assessed for eligibility, enrolled, allocated to each group, completing follow-up, and included in the final analysis.

Author response: Thank you. We revised the participant-flow description and Figure 2 to show the number approached/eligible (n=397), enrolled (n=384), allocated to the intervention and control groups (n=192 each), completing the six-month follow-up (n=192 each), and included in the final analysis (n=192 each).

Revised manuscript line(s): Fig. 2 (L312); L471–472

Comment 52 — Reviewer comment: • The Discussion could better distinguish the findings of the present study from findings reported in previous studies and explain possible reasons for similarities or differences.

Author response: Thank you. We revised the Discussion to distinguish the present study findings from previous research and added cautious explanations for similarities and differences, including contextual, educational, environmental, and product-related factors where relevant.

Revised manuscript line(s): L659–801

Comment 53 — Reviewer comment: • The Conclusion could be shortened slightly and focus mainly on the key findings, while emphasizing that confirmation in larger and multiple-site studies is needed.

Author response: Thank you. We shortened the Conclusion to focus on the key observed findings and the limitations of the single-site quasi-experimental design. It now emphasizes the need for larger, multi-site studies with stronger designs and repeated follow-up assessments to confirm the findings, assess generalizability, and determine whether changes are sustained and translate into clinical practice.

Revised manuscript line(s): L884–900

Reviewer #3:

Comment 54 — Reviewer comment: Reviewer #3: The manuscript is well-written and structured. The statistical analysis needs to be revised for in-depth understanding. An ANCOVA test can be added to the analysis for comparing the invention and control groups.

Author response: Thank you for this suggestion. We considered the recommendation. However, the Academic Editor explicitly indicated that the recommendation to add ANOVA/ANCOVA could be disregarded. We therefore retained the prespecified analyses and instead strengthened reporting of effect estimates, 95% confidence intervals, baseline imbalance, and design limitations.

Revised manuscript line(s): L421–452

Comment 55 — Reviewer comment: In line 100, “menstruation is often treated as a secretive and impure phenomenon” the wording “secretive and impure” needs to be improved

Author response: Thank you. We revised the sentence to use more neutral and culturally sensitive wording: menstruation is now described as being 'surrounded by secrecy and culturally embedded notions of impurity' rather than as a 'secretive and impure phenomenon.'

Revised manuscript line(s): L100–104

Comment 56 — Reviewer comment: In the method section, please clarify how 100% retention rate was achieved (line 343-345)

Author response: Thank you. We expanded the Data Collection Procedure to explain the retention process. Follow-up assessments were coordinated with scheduled academic activities, participant identification codes and attendance records were used to track participation, and students who missed intervention sessions were contacted through class representatives and offered make-up sessions. These procedures facilitated continued participation, and all 384 baseline participants completed the six-month follow-up.

Revised manuscript line(s): L407–415

Comment 57 — Reviewer comment: It is recommended to mention the IRB approval registration number and details.

Author response: Thank you. We added the name of the approving ethics/IRB body and the corresponding approval/registration number in the Methods ethics statement.

Revised manuscript line(s): L453–457

Comment 58 — Reviewer comment: Define PE (line 144)

Author response: Thank you. Participatory Education (PE) is now defined at its first relevant occurrence in the Methods as an interactive, learner-centered approach involving active engagement through discussion, reflection, role-play, practical demonstration, problem-solving, and peer learning. We also cite the WaterAid Menstrual Hygiene Matters training guide on which the participatory approach was based.

Revised manuscript line(s): L193–205; L219–246

Comment 59 — Reviewer comment: Relying on t-test is an outdated approach. It is recommended to perform ANCOVA test.

Author response: Thank you for the suggestion. As noted above, the Academic Editor specifically indicated that the recommendation to add ANOVA/ANCOVA could be disregarded. We therefore did not add ANCOVA and retained the prespecified statistical approach, while strengthening reporting of effect sizes, 95% confidence intervals, baseline differences, and the limitations of the non-randomized design.

Revised manuscript line(s): L421–452

Attachment Submitted filename: Response to Reviewers.docx

# Peer review: Decision Letter 1

**DOI:** 10.1371/journal.pone.0362603.r003

**Authors:** Alison Parker

27 Oct 2026

Effectiveness of a Participatory Educational Intervention on Menstrual Health and Hygiene Knowledge, Attitudes, and Practices Among Nursing and Midwifery Students in Bangladesh

PONE-D-26-40606R1

Dear Dr. Yamauchi,

We’re pleased to inform you that your manuscript has been judged scientifically suitable for publication and will be formally accepted for publication once it meets all outstanding technical requirements.

Within one week, you’ll receive an e-mail detailing the required amendments. When these have been addressed, you’ll receive a formal acceptance letter and your manuscript will be scheduled for publication.

An invoice will be generated when your article is formally accepted. Please note, if your institution has a publishing partnership with PLOS and your article meets the relevant criteria, all or part of your publication costs will be covered. Please make sure your user information is up-to-date by logging into Editorial Manager at Editorial Manager® and clicking the ‘Update My Information' link at the top of the page. For questions related to billing, please contact billing support.

If your institution or institutions have a press office, please notify them about your upcoming paper to help maximize its impact. If they’ll be preparing press materials, please inform our press team as soon as possible -- no later than 48 hours after receiving the formal acceptance. Your manuscript will remain under strict press embargo until 2 pm Eastern Time on the date of publication. For more information, please contact onepress@plos.org.

Kind regards,

Alison Parker

Academic Editor

PLOS One

Additional Editor Comments (optional):

Reviewers' comments:

Reviewer's Responses to Questions

**Comments to the Author**

Reviewer #2: (No Response)

**********

2. Is the manuscript technically sound, and do the data support the conclusions?

Reviewer #2: (No Response)

**********

3. Has the statistical analysis been performed appropriately and rigorously?

Reviewer #2: (No Response)

**********

4. Have the authors made all data underlying the findings in their manuscript fully available?

The PLOS Data policy

Reviewer #2: (No Response)

**********

5. Is the manuscript presented in an intelligible fashion and written in standard English?

Reviewer #2: (No Response)

**********

Reviewer #2: The authors have done an excellent job addressing the reviewers' comments and substantially revising the manuscript. The revisions have significantly improved the overall quality and clarity of the work. I appreciate their efforts and congratulate them on their successful revisions.

**********

what does this mean?). If published, this will include your full peer review and any attached files.

If you choose “no”, your identity will remain anonymous but your review may still be made public.

**Do you want your identity to be public for this peer review?**  For information about this choice, including consent withdrawal, please see our Privacy Policy

Reviewer #2: No

**********

# Editor report: Acceptance letter

**DOI:** 10.1371/journal.pone.0362603.r004

**Authors:** Alison Parker

PONE-D-26-40606R1

PLOS One

Dear Dr. Yamauchi,

I'm pleased to inform you that your manuscript has been deemed suitable for publication in PLOS One. Congratulations! Your manuscript is now being handed over to our production team.

At this stage, our production department will prepare your paper for publication. This includes ensuring the following:

* All references, tables, and figures are properly cited

* All relevant supporting information is included in the manuscript submission,

* There are no issues that prevent the paper from being properly typeset

You will receive further instructions from the production team, including instructions on how to review your proof when it is ready. Please keep in mind that we are working through a large volume of accepted articles, so please give us a few days to review your paper and let you know the next and final steps.

Lastly, if your institution or institutions have a press office, please let them know about your upcoming paper now to help maximize its impact. If they'll be preparing press materials, please inform our press team within the next 48 hours. Your manuscript will remain under strict press embargo until 2 pm Eastern Time on the date of publication. For more information, please contact onepress@plos.org.

You will receive an invoice from PLOS for your publication fee after your manuscript has reached the completed accept phase. If you receive an email requesting payment before acceptance or for any other service, this may be a phishing scheme. Learn how to identify phishing emails and protect your accounts at https://explore.plos.org/phishing.

If we can help with anything else, please email us at customercare@plos.org.

Thank you for submitting your work to PLOS One and supporting open access.

Kind regards,

PLOS One Editorial Office Staff

on behalf of

Dr. Alison Parker

Academic Editor

PLOS One
